# Asymmetric Synthesis and Biological Activity of Contact Pheromone of Western Flower Thrips, *Frankliniella occidentalis*

**DOI:** 10.3390/ijms252111699

**Published:** 2024-10-31

**Authors:** Chuanwen Lin, Wenya Zhu, Shuai Wu, Qinghua Bian, Jiangchun Zhong

**Affiliations:** 1 Department of Applied Chemistry, China Agricultural University, 2 West Yuanmingyuan Road, Beijing 100193, Chinabianqinghua@cau.edu.cn (Q.B.); 2 State Key Laboratory for Biology of Plant Diseases and Insect Pests, Institute of Plant Protection, Chinese Academy of Agricultural Sciences, Beijing 100193, China

**Keywords:** *Frankliniella occidentalis*, contact pheromone, biological activity, asymmetric synthesis

## Abstract

Western flower thrips, *Frankliniella occidentalis*, is a serious worldwide pest of agriculture and horticulture, and its contact pheromone is 7-methyltricosane. Two enantiomers of 7-methyltricosane were synthesized for the first time. The centra of our strategy were chiral auxiliaries to introduce stereocenter, and Wittig coupling to connect two blocks. The overall yields of our synthesis were 29–30% with seven steps. The electroantennogram (EAG) and the contact behavioral responses revealed that (*R*)-, (*S*)- and racemic 7-methyltricosane were separately bioactive, and the racemate was the most bioactive in the male arrestant activity and the female EAG test. This result provides valuable insights, showing that the racemate could be used for the support of the control of western flower thrips, which could be more easily prepared relative to more expensive enantiopure pheromone.

## 1. Introduction

**Figure 1:**
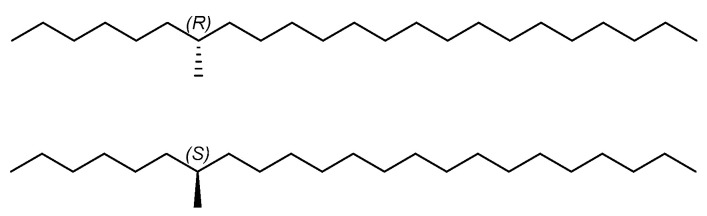
Two enantiomers of the contact pheromone of *F. occidentalis*.

Western flower thrips, *Frankliniella occidentalis* Pergande (Thysanoptera: Thripidae), is a serious worldwide pest of agriculture and horticulture originating from western North America [1,2]. This pest inhabits more than 250 crop species mainly including grains, vegetables, fruits, ornamentals and cotton, which has caused severe economic losses through feeding and oviposition on leaves, flowers and fruits [3,4]. In addition, it can transmit the plant-pathogenic viruses, such as tomato spotted wilt virus, impatiens necrotic spot virus and other *Tospovirus* species, to cause indirect damage to crops [5]. Long-term and frequent chemical insecticides use to control *F. occidentalis* has increased resistances to organophosphates, carbamates, pyrethroids and other pesticides [6,7]. Thus, it is meaningful to search for more environmentally friendly and efficient strategies [8,9]. In 2013, Kirk found that 7-methyltricosane could increase the contact time adult female and male *F. occidentalis* and identified it as a contact pheromone of *F. occidentalis* [10]. Moreover, 7-methyltricosane is the major component of hydrocarbons extracted from the mature female and male of the two-spot ladybird, *Adalia bipunctata*, which are necessary for mate recognition [11]. However, the bioactivity of two enantiomers of the contact pheromone of *F. occidentalis* has not been elucidated. Herein, we synthesized two enantiomers of 7-methyltricosane (Figure 1) and investigated the bioactivity of the contact pheromone.

## 2. Results and Discussion

### 2.1. Chemical Synthesis

#### 2.1.1. Synthesis of Chiral Intermediates (*R*)- and (*S*)-**6**

**Figure 2:**
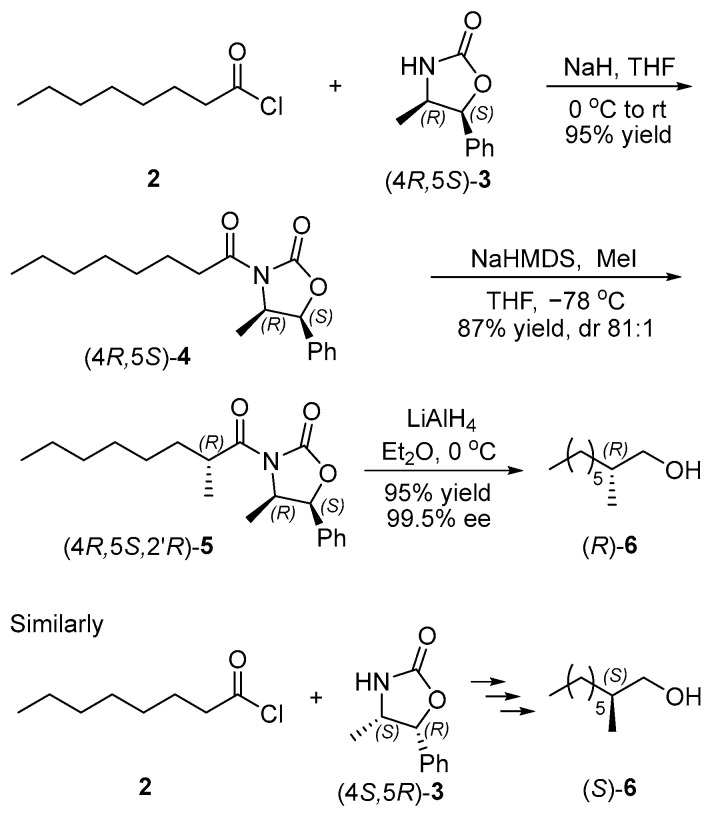
Synthesis of chiral intermediates (*R*)- and (*S*)-**6**.

Our synthesis began with preparations of chiral intermediates (*R*)- and (*S*)-**6** (Figure 2). The acylation of octanoyl chloride (**2**) with (4*R*,5*S*)-4-methyl-5-phenyl-2-oxazolidinon ((4*R*,5*S*)-**3**) catalyzed by NaH afforded oxazolidinone imide (4*R*,5*S*)-**4** in 95% yield [12], followed by a diastereoselective methylation with methyl iodide in the presence of sodium hexamethyldisilazide (NaHMDS) gave (4*R*,5*S*)-4-methyl-3-((*R*)-2-methyloctanoyl)-5-phenyloxazolidin-2-one ((4*R*,5*S*,2′*R*)-**5**) (87% yield). Its side reaction was the formation of the diastereoisomer of (4*R*,5*S*,2′*R*)-**5**, and dr was 81:1, determined by its ^13^C NMR spectra [13,14]. Then, methyl oxazolidinone imide (4*R*,5*S*,2′*R*)-**5** was converted into (*R*)-2-methyl-1-octanol ((*R*)-**6**) almost quantitatively via a reduction with LiAlH_4_ [15]. The specific rotation of chiral primary alcohols (*R*)-**6** was identical to the literature value [16], and the enantiomeric excesses (ee) was 95.5% determined by chiral HPLC of its 3,5-dinitrobenzoate (Figures S29 and S30) [17]. Similarly, acylation, diastereoselective methylation and reduction from (4*S*,5*R*)-4-methyl-5-phenyloxazolidin-2-one ((4*S*,5*R*)-**3**) and octanoyl chloride (**2**) provided (*S*)-2-methyloctan-1-ol ((*S*)-**6**) (80% yield, 95.0% ee, determined by chiral HPLC of its 3,5-dinitrobenzoate, Figures S29 and S31). 

#### 2.1.2. Synthesis of the Contact Pheromones (*R*)- and (*S*)-**1**

**Figure 3:**
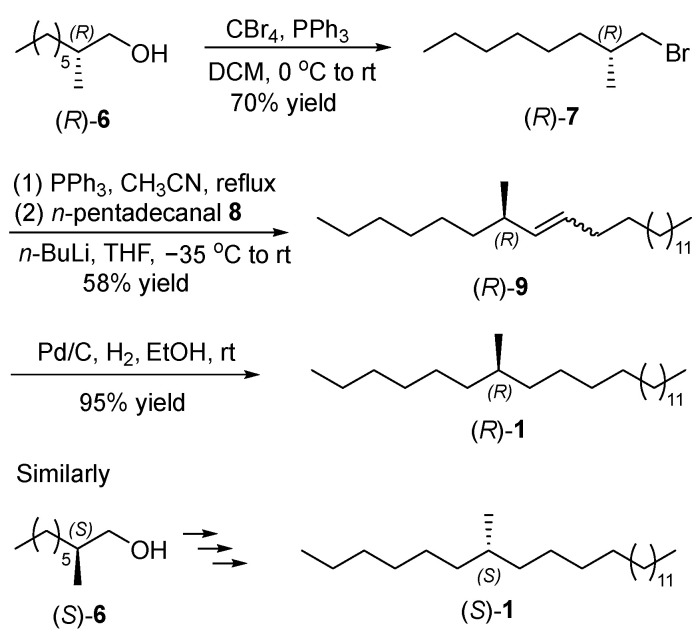
Synthesis of the contact pheromones (*R*)- and (*S*)-**1**.

With two key chiral intermediates (*R*)- and (*S*)-**6** in hand, the target contact pheromones (*R*)- and (*S*)-**1** were synthesized (Figure 3). Appel reaction of chiral alcohol (*R*)-**6** with tetrabromomethane and triphenylphosphine generated (*R*)-1-bromo-2-methyloctane ((*R*)-**7**) (70% yield) [18,19]. The phosphonium salt was then prepared through the reaction of hydrocarbon bromide (*R*)-**7** and triphenylphosphine [20]. The subsequent Wittig coupling with *n*-pentadecanal (**8**) afforded *Z*/*E* mixtures of (*R*)-7-methyltricos-8-ene ((*R*)-**9**) (58% yield, *Z*:*E* = 3.95:1, determined by its ^13^C NMR spectra) [21,22]. Its potential side reaction was base-catalyzed aldol condensation of aldehyde **8**. The final palladium-catalyzed hydrogenation converted chiral olefin (*R*)-**9** into (*R*)-7-methyltricosane ((*R*)-**1**) (95% yield) [23]. According to the similar procedure for pheromone (*R*)-**1**, (*S*)-7-methyltricosane ((*S*)-**1**) was synthesized from chiral primary alcohol (*S*)-**6** via bromination, Wittig coupling and catalytic hydrogenation. All synthetic products were characterized with ^1^H NMR, ^13^C NMR (Figures S1–S28), EIMS spectra. 

### 2.2. Biological Activity

#### 2.2.1. Behavioral Activity

In order to investigate the bioactivity of the contact pheromone of *F. occidentalis*, the contact behavioral responses of the adult females and males to three synthetic pheromones were tested (Table 1). The previous literature reported that adult *F. occidentalis* showed some strange mating behaviors. Adult females raised the abdomen up frequently to reject male mating attempts after their mating [24], and adult males wagged their abdomens sideways, which was often observed during male–male fighting [25]. The tests were performed as described in the previous literature [10]; a solution of (*R*)-, (*S*)- or racemic 7-methyltricosane in *n*-hexane was placed on a steel bead, while *n*-hexane was used as the control. The responses of each treatment were compared with the control (*n*-hexane) using an independent sample *T*-test. Adult females stayed in the vicinity of the coated bead longer (*p* < 0.05 for (*R*)-7-methyltricosane; *p* < 0.0001 for (*S*)- and racemic 7-methyltricosane) and raised their abdomens up more frequently (*p* < 0.05 for (*R*)-7-methyltricosane; *p* < 0.01 for (*S*)- and racemic 7-methyltricosane) after its initial contact. Adult males also stayed near the coated bead longer (*p* < 0.001 for (*R*)- and (*S*)-7-methyltricosane; *p* < 0.0001 for racemic 7-methyltricosane) and wagged the abdomen to the side more often (*p* < 0.001 for synthetic pheromones) after contact with the coated bead. The contact responses to three synthetic pheromones were then compared by Tukey’s pairwise comparisons (*p* < 0.05). Both adult females and males stayed near the bead coated with racemic 7-methyltricosane longer than that coated with (*R*)-7-methyltricosane, while they did not show significantly different behaviors, except that adult males wagged their abdomens sideways after contact with (*R*)-7-methylcosane and (*S*)-7-methyltricosane. Finaly, the differences between the males and females to three synthetic pheromones were compared by Tukey’s pairwise comparisons (*p* < 0.05). The adult males stayed near the coated bead longer for (*R*)- and *rac*-7-methylcosane. However, they did not show significant different arresting times for (*S*)-7-methyltricosane.

#### 2.2.2. Electroantennographic Activity

**Figure 4:**
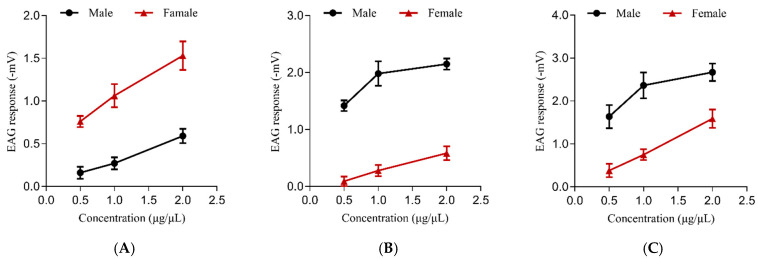
Electrophysiological responses to different concentration of synthetic pheromones from the antennas of the adult *F. occidentalis* males and females. Data in line charts are mean (±SEM, *n* = 5). EAG responses were obtained by subtracting EAG value of the solvent control from the results of antenna stimulation. (**A**) is the EAG responses to (*R*)-7-methyltricosane. (**B**) is the EAG responses to (*S*)-7-methyltricosane. (**C**) is the EAG responses to racemic 7-methyltricosane.

In order to further clarify the bioactivity of the contact pheromone of *F. occidentalis*, the electrophysiological responses to the synthetic pheromones from the antennas of the adult *F. occidentalis* were then measured by an electroantennogram (EAG) detection system and using the standard technique [26,27,28]. Figure 4 shows electrophysiological responses to different concentrations of synthetic pheromones from the antennas of the adult *F. occidentalis* males and females. (*R*)-, (*S*)- and racemic 7-methyltricosane all could elicit EAG response. Moreover, they all showed concentration-dependent responses to the incremental concentration from 0.5 to 2.0 μg/μL.

**Figure 5:**
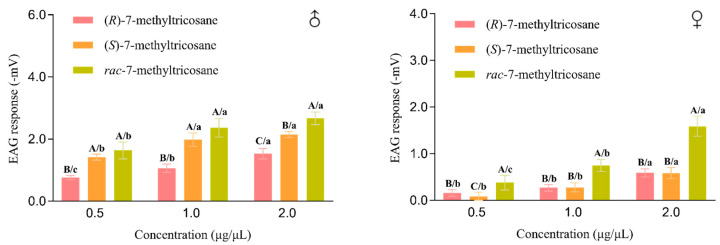
Mean EAG responses (±SEM, *n* = 5) of *F. occidentalis* females and males to (*R*)-, (*S*)- and racemic 7-methyltricosane. EAG responses were obtained by subtracting EAG value of the solvent control from the results of antenna stimulation. Bars with different letters indicate significant differences from each other (Tukey’s pairwise comparisons, *p* < 0.05). Capital letters (A–C) represent significant differences in three compounds of (*R*)-, (*S*)- and racemic 7-methyltricosane, while lowercase letters (a–c) represent significant differences in three concentrations of 0.5, 1.0 and 2.0 μg/μL.

The EAG responses to three synthetic pheromones were then compared by Tukey’s pairwise comparisons (*p* < 0.05) (Figure 5). Both female and males all showed significant response to three synthetic pheromones, and racemic 7-methyltricosane elicited the strongest responses from female and male antennas. Females showed no difference in response to (*R*)- and (*S*)-7-methyltricosane at two high concentrations of 1.0 and 2.0 μg/μL, whereas males showed significantly different responses to (*R*)- and (*S*)-7-methyltricosane at three concentrations.

Together with the contact behavioral responses and EAG tests, the bioactivity of contact pheromone of *F. occidentalis* were clarified. Two enantiomers of 7-methyltricosane and racemic 7-methyltricosane were separately bioactive against *F. occidentalis* males and females. Racemic 7-methyltricosane was more active than (*R*)- and (*S*)-7-methyltricosane in the male arrestant activity and the female EAG test. The previous literature confirmed that combined use of contact pheromone with a pathogen could enhance the infection of pest because contact pheromone has arrestant activity, which could increase the time of contact with pathogen [29]. Therefore, our results shed light on the fact that racemic 7-methyltricosane can be used for supporting the control of western flower thrips, and can be more easily prepared relative to more expensive enantiopure pheromone.

## 3. Materials and Methods

### 3.1. Chemical Synthesis

#### 3.1.1. General Information

Unless otherwise noted, all nonaqueous reactions were carried out under an argon atmosphere with Schlenk line. CH_2_Cl_2_, THF, Et_2_O and Et_3_N were distilled from CaH_2_ immediately prior to use. The reagents including NaH (60%), NaHMDS (99%), MeI (99%), LiAlH_4_ (97%), CBr_4_ (99%), PPh_3_ (99%) and *n*-BuLi (99%) were purchased and used as received. The 3,5-dinitrobenzoate derivatives of chiral primary alcohols (*R*)- and (*S*)-**6** were prepared according to Reference [17], and details can be seen in the Supplementary Materials (Scheme S1, page S2). The measurement of enantiomeric excesses (ee) was performed on an Agilent 1200 HPLC Series (Santa Clara, CA, USA) system with a Daicel Chiralcel OJ-H column. Optical rotations were determined by a Rudolph AUTOPOL-IV polarimeter (Flanders, NY, USA) at 25 °C. ^1^H and ^13^C NMR spectra were recorded at 500 and 125 MHz on a Bruker Ascend^TM^ 500MHz spectrometer (Billerica, MA, USA), respectively. Tetramethylsilane (0.00 ppm) was used as internal standard for ^1^H NMR and CDCl_3_ (77.16 ppm) for ^13^C NMR. High-resolution mass (HRMS) data were taken on a Waters LCT Premier™ with an ESI mass spectrometer (Milford, CT, USA).

#### 3.1.2. Synthesis of Chiral Intermediates (*R*)- and (*S*)-**6**

(4*R*,5*S*)-4-methyl-3-octanoyl-5-phenyloxazolidin-2-one ((4*R*,5*S*)-**4**) (CAS 1341177-96-5)

(4*R*,5*S*)-4-Methyl-5-phenyloxazolidin-2-one ((4*R*,5*S*)-**3**) (7.58 g, 42.81 mmol) was dissolved in THF (86 mL) at room temperature. The solution was cooled to 0 °C, then NaH (2.60 g, 60% suspension in mineral oil, 65.00 mmol) was added in portions. The resulting mixture was allowed to warm to room temperature and stirred for 2 h. The temperature dropped to 0 °C, followed by the addition of octanoyl chloride (10.44 g, 64.20 mmol) via a syringe. After the reaction mixture was allowed to warm to room temperature and stirred for 12 h, monitored by thin-layer chromatography (petroleum ether/ethyl acetate = 5:1), it was quenched with saturated aqueous NH_4_Cl (35 mL) at 0 °C. The organic phase was separated, and the aqueous phase was extracted with ethyl acetate (20 mL × 3). The combined ester extracts and the organic phase were washed with saturated brine (40 mL), dried over anhydrous Na_2_SO_4_, and concentrated under reduced pressure. The final column chromatography on silica gel (petroleum ether/ethyl acetate = 30:1) afforded (4*R*,5*S*)-4-methyl-3-octanoyl-5-phenyloxazolidin-2-one ((4*R*,5*S*)-**4**) (12.33 g, 95% yield) as a white solid. R_f_ = 0.33 (petroleum ether/ethyl acetate = 5:1). [α]_D_^25^ = +34.9 (c 6.31, CHCl_3_). ^1^H NMR (500 MHz, CDCl_3_) δ 7.38–7.30 (m, 3H), 7.25–7.22 (m, 2H), 5.61 (d, *J* = 7.3 Hz, 1H), 4.71 (p, *J* = 6.7 Hz, 1H), 2.96–2.81 (m, 2H), 1.67–1.58 (m, 2H), 1.33–1.24 (m, 8H), 0.85–0.82 (m, 6H). ^13^C NMR (126 MHz, CDCl_3_) δ 173.31, 153.16, 133.52, 128.84, 128.80, 125.76, 79.05, 54.84, 35.74, 31.78, 29.18, 29.15, 24.43, 22.71, 14.67, 14.18. HRMS (ESI) calcd for C_18_H_26_O_3_N [M + H]^+^ 304.19072, found 304.19053.

(4*R*,5*S*)-4-methyl-3-((*R*)-2-methyloctanoyl)-5-phenyloxazolidin-2-one ((4*R*,5*S*,2′*R*)-**5**) (new compound)

(4*R*,5*S*)-4-Methyl-3-octanoyl-5-phenyloxazolidin-2-one ((4*R*,5*S*)-**4**) (5.00 g, 16.49 mmol) was dissolved in THF (100 mL) at room temperature. The solution was cooled to −78 °C, then NaHMDS (12.38 mL, 2.0 M in THF, 24.76 mmol) was added slowly via a syringe. After the resulting mixture was stirred for 2.5 h, MeI (11.71 g, 82.50 mmol) was added slowly via a syringe over 4 h at the same temperature. The reaction mixture was stirred for an additional 4 h at −78 °C, monitored by thin-layer chromatography (petroleum ether/ethyl acetate = 5:1). The reaction was quenched with saturated aqueous NH_4_Cl (40 mL). The organic phase was separated, and the aqueous phase was extracted with ethyl acetate (20 mL × 3). The combined ester extracts and the organic phase were washed with saturated brine (60 mL), dried over anhydrous Na_2_SO_4_ and concentrated under reduced pressure. The final column chromatography on silica gel (petroleum ether/ethyl acetate = 40:1) afforded (4*R*,5*S*)-4-methyl-3-((*R*)-2-methyloctanoyl)-5-phenyloxazolidin-2-one ((4*R*,5*S*,2′*R*)-**5**) (4.56 g, 87% yield, dr 81:1 determined by its ^13^C NMR spectra) as a white solid. R_f_ = 0.25 (petroleum ether/ethyl acetate = 5:1). [α]_D_^25^ = +9.05 (c 1.33, CHCl_3_). ^1^H NMR (500 MHz, CDCl_3_) δ 7.43–7.36 (m, 3H), 7.32–7.30 (m, 2H), 5.66 (d, *J* = 7.2 Hz, 1H), 4.77 (p, *J* = 6.7 Hz, 1H), 3.72 (dt, *J* = 6.9 Hz, 1H), 1.77–1.71 (m, 1H), 1.44–1.40 (m, 1H), 1.32–1.26 (m, 8H), 1.19 (d, *J* = 6.8 Hz, 3H), 0.90–0.87 (m, 6H). ^13^C NMR (126 MHz, CDCl_3_) δ 177.30, 152.84, 133.57, 128.87, 128.85, 125.78, 78.94, 55.07, 37.97, 33.69, 31.86, 29.46, 27.38, 22.75, 17.29, 14.57, 14.22. HRMS (ESI) calcd for C_19_H_26_O_3_N [M − H]^+^ 316.19072, found 316.19055.

(*R*)-2-methyloctan-1-ol ((*R*)-**6**) (CAS 120925-50-0)

LiAlH_4_ (0.72 g, 18.97 mmol) was placed in a 100 mL Schlenk flask at room temperature, and anhydrous Et_2_O (25 mL) was added. The resulting suspension was cooled to 0 °C, then (4*R*,5*S*)-4-methyl-3-((*R*)-2-methyloctanoyl)-5-phenyloxazolidin-2-one ((4*R*,5*S*, 2′*R*)-**5**) (4.00 g, 12.61 mmol) was added. The reaction mixture was stirred for 2 h at 0 °C and monitored by thin-layer chromatography (petroleum ether/ethyl acetate = 5:1), followed by the neutralization with dilute hydrochloric acid (20 mL, 1.0 M, 20.0 mmol). The organic phase was separated, and the aqueous phase was extracted with Et_2_O (15 mL × 3). The combined ether extracts and the organic phase were washed sequentially with saturated aqueous NaHCO_3_ (10 mL) and saturated brine (15 mL), dried over anhydrous Na_2_SO_4_ and concentrated. The final column chromatography on silica gel (*n*-pentane/ether = 10:1) afforded (*R*)-2-methyloctan-1-ol ((*R*)-**6**) (1.45 g, 80% yield, 99.5% ee, determined by chiral HPLC of its 3,5-dinitrobenzoate) as a colorless liquid. R_f_ = 0.51 (petroleum ether/ethyl acetate = 5:1). [α]_D_^25^ = +7.69 (c 3.64, CHCl_3_) Lit. [16] [α]_D_^22^ = +5.0 (c 1.2, CHCl_3_). ^1^H NMR (500 MHz, CDCl_3_) δ 3.46 (dd, *J* = 10.5, 5.8 Hz, 1H), 3.37 (dd, *J* = 10.5, 6.6 Hz, 1H), 1.61–1.52 (m, 1H), 1.42 (br s, 1H), 1.38–1.21 (m, 9H), 1.08–1.02 (m, 1H), 0.87 (d, *J* = 6.8 Hz, 3H), 0.83 (t, *J* = 7.1 Hz, 3H). ^13^C NMR (126 MHz, CDCl_3_) δ 68.55, 35.90, 33.29, 32.00, 29.74, 27.08, 22.80, 16.71, 14.23. HRMS (ESI) calcd for C_9_H_19_O [M − H]^+^ 143.14304, found 143.14310.

(4*S*,5*R*)-4-methyl-3-octanoyl-5-phenyloxazolidin-2-one ((4*S*,5*R*)-**4**) (CAS 1341177-89-6)

According to the similar procedure for oxazolidinone imide (4*R*,5*S*)-**4**, the acylation of (4*S*,5*R*)-4-methyl-5-phenyloxazolidin-2-one((4*S*,5*R*)-**3**) (10.00 g, 56.47 mmol) with octanoyl chloride (13.75 g, 84.52 mmol) afforded (4*S*,5*R*)-4-methyl-3-octanoyl-5-phenyloxazolidin-2-one ((4*S*,5*R*)-**4**) (16.26 g, 95% yield) as a white solid. R_f_ = 0.33 (petroleum ether/ethyl acetate = 5:1). [α]_D_^25^ = −36.4 (c 1.98, CHCl_3_). ^1^H NMR (500 MHz, CDCl_3_) δ 7.38–7.30 (m, 3H), 7.27–7.22 (m, 2H), 5.61 (d, *J* = 7.3 Hz, 1H), 4.72 (p, *J* = 6.7 Hz, 1H), 2.96–2.67 (m, 2H), 1.67–1.58 (m, 2H), 1.34–1.22 (m, 8H), 0.85–0.83 (m, 6H). ^13^C NMR (126 MHz, CDCl_3_) δ 173.32, 153.16, 133.53, 128.84, 128.81, 125.76, 79.05, 54.84, 35.74, 31.79, 29.18, 29.15, 24.43, 22.72, 14.68, 14.19, 1.13. HRMS (ESI) calcd for C_18_H_25_NO_3_Na [M + Na]^+^ 326.17266, found 326.17147.

(4*S*,5*R*)-4-methyl-3-((*S*)-2-methyloctanoyl)-5-phenyloxazolidin-2-one ((4*S*,5*R*,2*S*)-**5**) (new compound)

According to the similar procedure for methyl oxazolidinone imide (4*R*,5*S*,2′*R*)-**5**, diastereoselective methylation of (4*S*,5*R*)-4-methyl-3-octanoyl-5-phenyloxazolidin-2-one ((4*S*,5*R*)-**4**) (5.00 g, 16.49 mmol) with MeI (11.71 g, 82.51 mmol) afforded (4*S*,5*R*)-4-methyl-3-((*S*)-2-methyloctanoyl)-5-phenyloxazolidin-2-one((4*S*,5*R*,2′*S*)-**5**) (4.18 g, 80% yield, dr 83:1 determined by its ^13^C NMR spectra) as a white solid. R_f_ = 0.25 (petroleum ether/ethyl acetate = 5:1). [α]_D_^25^ = −10.00 (c 0.88, CHCl_3_). ^1^H NMR (500 MHz, CDCl_3_) δ 7.43–7.35 (m, 3H), 7.32–7.30 (m, 2H), 5.66 (d, *J* = 7.2 Hz, 1H), 4.77 (p, *J* = 6.6 Hz, 1H), 3.72 (dt, *J* = 6.6 Hz, 1H), 1.75–1.71 (m, 1H), 1.44–1.38 (m, 1H), 1.32–1.26 (m, 8H), 1.19 (d, *J* = 6.8 Hz, 3H), 0.90–0.87 (m, 6H). ^13^C NMR (126 MHz, CDCl_3_) δ 177.23, 152.79, 133.55, 128.81, 128.80, 125.74, 78.88, 55.02, 37.92, 31.82, 29.41, 27.33, 22.70, 17.25, 14.52, 14.18. HRMS (ESI) calcd for C_19_H_28_NO_3_ [M + H]^+^ 318.20637, found 318.20835.

(*S*)-2-methyloctan-1-ol ((*S*)-**6**) (CAS 116013-69-5)

According to the similar procedure for chiral alcohol (*R*)-**6**, the reduction of (4*S*,5*R*)-4-methyl-3-((*S*)-2-methyloctanoyl)-5-phenyloxazolidin-2-one ((4*S*,5*S*,2*R*)-**5**) (4.00 g, 12.61 mmol) with LiAlH_4_ (0.72 g, 18.97 mmol) afforded (*S*)-2-methyloctan-1-ol ((*S*)-**6**) (1.45 g, 80% yield, 95% ee, determined by chiral HPLC of its 3,5-dinitrobenzoate) as a colorless liquid. R_f_ = 0.51 (petroleum ether/ethyl acetate = 5:1). [α]_D_^25^ = −9.0 (c 3.12, CHCl_3_) Lit. [30] [α]_D_^22^ = −9.0 (c 1.0, EtOH). ^1^H NMR (500 MHz, CDCl_3_) δ 3.46 (dd, *J* = 10.5, 5.8 Hz, 1H), 3.37 (dd, *J* = 10.5, 6.6 Hz, 1H), 1.61–1.52 (m, 1H), 1.41 (br s, 1H), 1.38–1.21 (m, 9H), 1.08–1.02 (m, 1H), 0.86 (d, *J* = 6.7 Hz, 3H), 0.84 (t, *J* = 6.4 Hz, 3H). ^13^C NMR (126 MHz, CDCl_3_) δ 68.55, 35.90, 33.29, 29.74, 27.08, 22.80, 16.72, 14.23. HRMS (ESI) calcd for C_9_H_20_O [M]^+^ 144.15087, found 144.15030.

#### 3.1.3. Synthesis of the Contact Pheromones (*R*)- and (*S*)-**1**

(*R*)-1-bromo-2-methyloctane ((*R*)-**7**) (163530-44-7)

Triphenylphosphine (1.55 g, 5.91 mmol) was dissolved in CH_2_Cl_2_ (7.3 mL) at room temperature. The solution was cooled to 0 °C, then CBr_4_ (1.23 g, 3.71 mmol) were added. The reaction mixture was allowed to warm to room temperature and stirred for 30 min. The temperature dropped to 0 °C, followed by the addition of (*R*)-2-methyloctan-1-ol ((*R*)-**6**) (0.50 g, 3.47 mmol) in CH_2_Cl_2_ (5.7 mL) via a syringe. After the reaction mixture was stirred for 12 h at the same temperature and monitored by thin-layer chromatography (petroleum ether), the solvent was removed. The final column chromatography on silica gel (petroleum ether/ethyl acetate = 100:1) afforded (*R*)-1-bromo-2-methyloctane ((*R*)-**7**) (0.50 g, 70% yield) as a colorless oil. R_f_ = 0.86 (petroleum ether). [α]_D_^25^ = −2.11 (c 1.90, CHCl_3_). ^1^H NMR (500 MHz, CDCl_3_) δ 3.40 (dd, *J* = 9.8, 5.0 Hz, 1H), 3.32 (dd, *J* = 9.8, 6.2 Hz, 1H), 1.83–1.74 (m, 1H), 1.46–1.41 (m, 1H), 1.31–1.19 (m, 9H), 1.01 (d, *J* = 6.6 Hz, 3H), 0.89 (t, *J* = 6.8 Hz, 3H). ^13^C NMR (126 MHz, CDCl_3_ δ 41.77, 35.36, 35.03, 31.94, 29.52, 26.99, 22.78, 18.94, 14.23. HRMS (ESI) calcd for C_9_H_19_Br [M]^+^ 206.06646, found 206.06714.

(*R*)-7-methyltricos-8-ene ((*R*)-**9**) (new compound)

Triphenylphosphine (0.72 g, 2.75 mmol) was dissolved in acetonitrile (7 mL) at room temperature. Then, (*R*)-1-bromo-2-methyloctane ((*R*)-**7**) (0.38 g, 1.84 mmol) in acetonitrile (6 mL) was added via a syringe. After the reaction solution was heated to reflux and stirring for 72 h, the solvent was removed under reduced pressure. The final column chromatography on silica gel (dichloromethane/methanol = 10:1) afforded (*R*)-2-methyl-1-octanyl triphenylphosphonium bromide (0.46 g) as a pale yellow solid.

(*R*)-2-Methyl-1-octanyl triphenylphosphonium bromide (0.10 g, 0.21 mmol) and THF (1 mL) were added in a 25 mL Schlenk tube at room temperature. The temperature dropped to −35 °C, followed by a cautious addition of *n*-butyl lithium solution (0.27 mL, 2.4 M in *n*-hexane, 0.65 mmol) via a syringe. The resulting mixture was allowed to warm to room temperature and stirred for 15 min. After the mixture was cooled −35 °C, *n*-pentadecanal (**8**) (0.11 g, 0.49 mmol) in THF (0.25 mL) was added dropwise. The temperature raised to room temperature again and stirred for additional 6 h, monitored by thin-layer chromatography (petroleum ether). The reaction mixture was cooled to 0 °C and quenched with saturated aqueous NH_4_Cl (5 mL). The organic phase was separated, and the aqueous phase was extracted with Et_2_O (3 × 4 mL). The combined ether extracts and the organic phase were washed with saturated aqueous NaCl (6 mL), dried over anhydrous Na_2_SO_4_ and concentrated under reduced pressure. The final column chromatography on silica gel (petroleum ether/ethyl acetate = 100:1) afforded the *Z*/*E* mixtures of (*R*)-7-methyltricos-8-ene ((*R*)-**9**) (0.041 g, 58% yield, *Z*:*E* = 3.95:1, determined by its ^13^C NMR spectra) as a colorless oil. R_f_ = 0.89 (petroleum ether). [α]_D_^25^ = +5.3 (c 1.50, CHCl_3_). ^1^H NMR (500 MHz, CDCl_3_) δ 5.24–5.20 (m, 1H), 5.05–5.00 (m, 1H), 2.34–2.29 (m, 1H), 1.96–1.92 (m, 2H), 1.20–1.17 (m, 34H), 0.84 (d, *J* = 6.7 Hz, 3H), 0.82–0.80 (m, 6H). ^13^C NMR (126 MHz, CDCl_3_) δ 136.57, 128.51, 37.78, 37.78, 32.10, 31.79, 30.11, 29.87, 29.83, 29.74, 29.67, 29.53, 29.52, 27.65, 22.86, 21.60,14.28. HRMS (ESI) calcd for C_24_H_48_ [M]^+^ 336.37505, found 336.37585.

(*R*)-7-methyltricosane ((*R*)-**1**) (new compound)

Ten percent palladium on carbon (0.0090 g) was placed in a 25 mL Schlenk tube at room temperature, and hydrogen was charged. (*R*)-7-Methyltricos-8-ene ((*R*)-**9**) (0.060 g, 0.178 mmol) in ethanol (5 mL) was then added. After the reaction mixture was maintained for 12 h under a hydrogen atmosphere and monitored by thin-layer chromatography (petroleum ether), it was filtered. The filter was washed with *n*-hexane (30 mL) and was combined with the filtrate. The solvent was removed under reduced pressure. The final column chromatography on silica gel (petroleum ether) afforded (*R*)-7-methyltricosane ((*R*)-**1**) (0.057 g, 95% yield) as a white solid. R_f_ = 0.89 (petroleum ether). [α]_D_^25^ = +0.17 (c 2.31, CHCl_3_). ^1^H NMR (500 MHz, CDCl_3_) δ 1.36 –1.31 (m, 1H), 1.27–1.24 (m, 40H), 0.83 (t, *J* = 6.8 Hz, 6H), 0.79 (d, *J* = 6.6 Hz, 3H). ^13^C NMR (126 MHz, CDCl_3_) δ 37.28, 32.93, 32.14, 32.10, 30.21, 29.91, 29.88, 29.84, 29.54, 27.26, 27.23, 22.87, 19.88, 14.28. EIMS (*m*/*z* (%)): 338.4 (0.3, M^+^), 253.3(12), 112.1 (43), 99.1 (12), 85.1 (37), 71.1 (94), 57.1 (100), 43.1 (46).

(*S*)-1-bromo-2-methyloctane ((*S*)-**7**) (141622-95-9)

According to the similar procedure for chiral bromide (*R*)-**7**, Appel reaction of (*S*)-2-methyloctan-1-ol ((*S*)-**6**) (0.60 g, 4.16 mmol) with triphenylphosphine (3.10 g, 11.81 mmol) and CBr_4_ (2.46 g, 7.42 mmol) afforded (*S*)-1-bromo-2-methyloctane ((*S*)-**7**) (0.79 g, 92% yield) as a colorless oil. R_f_ = 0.86 (petroleum ether). [α]_D_^25^ = +1.37(c 2.91, CHCl_3_) ^1^H NMR (500 MHz, CDCl_3_) δ 3.40 (dd, *J* = 9.7, 4.9 Hz, 1H), 3.32 (dd, *J* = 9.8, 6.2 Hz, 1H), 1.81–1.75 (m, 1H), 1.46–1.40 (m, 1H), 1.31–1.23 (m, 9H), 1.01 (d, *J* = 6.6 Hz, 3H), 0.89 (t, *J* = 6.6 Hz, 3H). ^13^C NMR (126 MHz, CDCl_3_) δ 41.78, 35.36, 35.03, 31.94, 29.52, 26.99, 22.78, 18.95, 14.24. HRMS (ESI) calcd for C_9_H_19_Br [M]^+^ 206.06646, found 206.06700.

(*S*)-7-methyltricos-8-ene ((*S*)-**9**) (new compound)

According to the similar procedure for chiral alkene (*R*)-**9**, Wittig reaction of *n*-pentadecanal (**8**) (0.11 g, 0.49 mmol) with (*S*)-2-methyl-1-octanyl triphenylphosphonium bromide (0.10 g, 0.21 mmol) derived from (*S*)-1-bromo-2-methyloctane (*S*)-**7**) and triphenylphosphine afforded the *Z*/*E* mixtures of (*S*)-7-methyltricos-8-ene ((*S*)-**9**) (0.038 g, 54% yield, *Z*:*E* = 3.10:1, determined by its ^13^C NMR spectra) as a colorless oil. R_f_ = 0.89 (petroleum ether). [α]_D_^25^ = −1.6 (c 2.57, CHCl_3_). ^1^H NMR (500 MHz, CDCl_3_) δ 5.28–5.21(m, 1H), 5.09–5.04 (m, 1H), 2.40–2.34 (m, 1H), 2.00–1.97 (m, 2H), 1.23–1.20 (m, 34H), 0.88 (d, *J* = 6.7 Hz, 3H), 0.86–0.83 (m, 6H). ^13^C NMR (126 MHz, CDCl_3_) δ 136.58, 128.51, 37.77, 32.09, 31.78, 30.11, 29.86, 29.82, 29.73, 29.66, 29.52, 27.64, 22.85, 21.59, 14.27. HRMS (ESI) calcd for C_24_H_48_ [M]^+^ 336.37505, found 336.37583.

(*S*)-7-methyltricosane ((*S*)-**1**) (CAS 55193-71-0)

According to the similar procedure for sex pheromone (*R*)-**1**, palladium-catalyzed hydrogenation of (*S*)-7-methyltricos-8-ene ((*S*)-**9**) (0.023 g, 0.068 mmol) afforded (*S*)-7-methyltricosane ((*S*)-**1**) (0.022 g, 96% yield) as a white solid. R_f_ = 0.89 (petroleum ether). [α]_D_^25^ = −2.74 (c 1.31, CHCl_3_). ^1^H NMR (500 MHz, CDCl_3_) δ 1.30–1.28 (m, 1H), 1.25 –1.14 (m, 40H), 0.81 (t, *J* = 6.8 Hz, 6H), 0.77 (d, *J* = 6.6 Hz, 3H). ^13^C NMR (126 MHz, CDCl_3_) δ 37.27, 32.92, 32.13, 32.09, 30.20, 29.89, 29.86, 29.53, 27.25, 27.21, 22.86, 19.88, 14.28. EIMS (*m*/*z*(%)): 338.4 (0.2, M^+^), 253.3 (10), 112.1 (41), 85.1 (36), 71.1 (93), 57.1 (100), 43.1 (47).

### 3.2. Insects

The original colony of *Frankliniella occidentalis* was obtained from the Chinese Academy of Agricultural Sciences (Beijing, China). The thrips were reared on pods of fresh beans (*Phaseolus vulgaris*) in glass gars (0.5 L), which were kept in a rearing room at 25 ± 1 °C, 50–60% relative humidity and a photoperiod of 14 h light/10 h dark. The adult sex ratio (female/male) was 10:1, which was similar to the ratio (60–90% females) at high densities of thrips within a greenhouse [31] and the ratio (0–10% males) in apple bud clusters during early bloom [32]. The adults were collected individually with an aspirator before bioassay.

### 3.3. Behavioral Activity

According to the similar procedure of Kirk’s report [10], the contact behavioral responses of adult *F. occidentalis* to three synthetic pheromones were studied. Racemic 7-methyltricosane, (*R*)- and (*S*)-7-methyltricosane were test compounds, while *n*-hexane was used as the control. The test compound was dissolved in *n*-hexane and diluted to a working concentration of 200 pg/μL. A solution of the test compound (5 μL) was placed on a clean steel bead (diam. 3 mm), which was put in a clean glass Petri dish (diam. 85 mm). The individual thrip was then placed near the coated bead, and the response was observed for 3 min after its initial contact with the bead. The time taken to leave a 5 mm zone around the bead, the number of times females raised their abdomens up to an at least 45° angle to the substrate and the number of times males wagged their abdomens sideways at above a 20° angle to the side were recorded. The dish and steel bead were replaced after every five thrips were tested. Each adult *F. occidentalis* was tested once, and 20 adult males and 20 adult females were used for each treatment. All behavioral tests were performed at 25 ± 1 °C, 50–60% relative humidity and a photoperiod of 14 h light/10 h dark from 12:00 to 16:00.

### 3.4. Electroantennographic Activity

The electrophysiological responses to the synthetic pheromones from the antennas of the adult *F. occidentalis* were measured by an electroantennogram detection system, which comprises an interface box (IDAC-4, Syntech, Kirchzaeten, Germany) with electrodes (silver–silver chloride, WPI, Sarasota, FL, USA) contained within electrolyte-filled glass capillary (0.1 mol/L KCl solution), a stimulus controller (CS-55 model, Syntech, Kirchzaeten, Germany) and a computer with Syntech EAG software (EAGPro v2.0). Racemic 7-methyltricosane, (*R*)- and (*S*)-7-methyltricosane were dissolved in *n*-hexane respectively and diluted to three concentrations of 2.0 μg/μL, 1.0 μg/μL and 0.5 μg/μL. A solution of the pheromone (2.5 μL) was added to a piece of filter paper (0.5 × 5 cm), then transferred it to a glass Pasteur pipette (15.0 cm long) as a stimulus. Filter paper soaked with *n*-hexane (2.5 μL) acted as the solvent control stimulus [26]. The head of adult *F. occidentalis* was excised from the body and connected to a reference electrode; then, two antennas were linked with a recording electrode [27]. The tip of Pasteur pipette was inserted into the hole of the main airstream tube about 20 cm from the antenna. The antennal preparation was presented with a continuous charcoal-filtered and humidified airstream (800 mL/min). Stimuli were provided by puffing a 0.2 s pulse of air (10 mL/s) from Pasteur pipette into the main airstream. At least 30 s between each stimulation was allowed for the antenna to stabilize. The EAG signal was amplified with a 10 × AC/DC preamplifier (Syntech, Kirchzarten, Germany) and recorded with a PC-based EAG software (EAGPro v2.0, Syntech). Each trial was conducted in the sequence of *n*-hexane, test pheromone and *n*-hexane, and repeated five times with the same antenna. Five male and five female antennas were used for each treatment. EAG value of the solvent control was obtained from the mean value of the two measurements, and corrected EAG responses were obtained by subtracting EAG value of the solvent control from the results of antenna stimulation [28]. EAG tests were conducted at 25 ± 1 °C, 85% relative humidity and a photoperiod of 14 h light/10 h dark.

### 3.5. Statistical Analysis

The normality and homogeneity of variance were checked prior to statistical analysis of data. The difference of contact behavioral responses between each treatment with control (*n*-hexane) were analyzed by independent sample *t*-test. The contact behavioral responses among the three synthetic pheromones were analyzed using one-way ANOVA, followed by Tukey’s HSD test (*p* < 0.05), as well as the comparisons between males and females. For the EAG tests, the data were also analyzed by one-way ANOVA, followed by Tukey’s HSD test (*p* < 0.05). All statistical calculations were conducted using SPSS Statistics version 27 from IBM Corporation (New York, NY, USA).

## 4. Conclusions

In summary, we have achieved asymmetric synthesis of two enantiomers of the contact pheromone of *F. occidentalis* for the first time. The key steps to our strategy involved chiral auxiliaries to introduce stereocenter, and Wittig coupling to connect two blocks. Moreover, we have clarified bioactivity of the contact pheromone via the contact behavioral responses and EAG tests. (*R*)-, (*S*)- and racemic 7-methyltricosane were separately bioactive and the racemate was the most bioactive in the male arrestant activity and the female EAG test. Our results shed light on that racemic 7-methyltricosane could be used for the support of the control of western flower thrips. Furthermore, compared with the asymmetric synthesis of (*R*)- and (*S*)-7-methyltricosane, the preparation of the racemic 7-methyltricosane has lower costs and energy consumption, greater atom economy and is more environmentally friendly.

## Figures and Tables

**Table 1:** Behavioral responses of adult *F. occidentalis* males and females to synthetic pheromones and control after contact with coated steel beads.

	Treatment			Control
	(*R*)-7-Methyltricosane ^1^	(*S*)-7-Methyltricosane ^1^	*rac*-7-Methyltricosane ^1^	*n*-Hexane
Females				
Median time in vicinity (s) ^2^	38.8 (10, 174) *^,b,A^	92.9 (41, 180) ****^,ab,A^	103.9 (24, 180) ****^,a,A^	2.6 (1, 4) ^4^
Median no. of abdomen raises ^3^	4.5 (0, 18) *^,a^	9.7 (2, 46) **^,a^	8.5 (2, 31) **^,a^	0.2 (0, 2)
Males				
Median time in vicinity (s) ^2^	85 (15, 160) ***^,b,B^	87.3 (15, 180) ***^,b,A^	160.8 (83, 180) ****^,a,B^	1.6 (1, 3)
Median no. of abdomen wags ^3^	1.9 (0, 4) ***^,b^	7 (1, 20) ***^,a^	3.4 (1, 10) ***^,b^	0.1 (0, 1)

^1^ Working concentration is 200 pg/μL and solvent is *n*-hexane. ^2^ Time taken to leave a 5 mm zone around the coated bead from initial contact. ^3^ Within 3 min after initial contact with the coated bead. ^4^ Median is the median in brackets (95% confidence intervals, *n* = 20). Significance level of independent sample *t*-test between each treatment and the control: * *p* < 0.05; ** *p* < 0.01; *** *p* < 0.001; **** *p* < 0.0001. Medians with different lowercase letters (a and b) indicate significant differences (Tukey’s pairwise comparisons, *p* < 0.05) in three compounds of (*R*)-, (*S*)- and racemic 7-methyltricosane, while capital letters (A and B) indicate significant differences (Tukey’s pairwise comparisons, *p* < 0.05) between the males and females.

## Data Availability

The data presented in this article are available in the Supplementary Materials.

## Supplementary Materials

The following supporting information can be downloaded at: https://www.mdpi.com/article/10.3390/ijms252111699/s1.
