# Supplementary material for: Asymmetric Synthesis and Biological Activity of Contact Pheromone of Western Flower Thrips, Frankliniella occidentalis
Source: Int J Mol Sci. 2024 Oct 31;25(21):11699. doi: 10.3390/ijms252111699 (PMC11546309; doi:10.3390/ijms252111699)
Supplement: Supplementary file 1 [file ijms-25-11699-s001.zip › ijms-3249356-supplementary.pdf]

SUPPORTING INFORMATION

Asymmetric synthesis and biological activity of contact pheromone of western flower thrips, *Frankliniella occidentalis*

Chuanwen Lin,[1] Wenya Zhu,[1] Shuai Wu,[2] Qinghua Bian[1] and Jiangchun Zhong[1*]

[1] Department of Applied Chemistry, China Agricultural University, 2 West Yuanmingyuan Road, Beijing 100193, China.

[2] State Key Laboratory for Biology of Plant Diseases and Insect Pests, Institute of Plant Protection, Chinese Academy of Agricultural Sciences, Beijing 100193, China.

* Correspondence: zhong@cae.edu.cn; Tel.: +8601062731356

Table of Contents

1. General Information……………………………………………………………………………...S2

2. Synthesis of 3,5-dinitrobenzoates (*R*)- and (*S*)-**11**……………………………………………………S2

3. ^{1}H, ^{13}C NMR spectra of the products……………………………………………………………S4

4. HPLC chromatography of the compounds………………………………………………………S18

5. References……………………………………………………………………………………………S19

1. General information

Unless otherwise noted, all nonaqueous reactions were carried out under an argon atmosphere with Schlenk line. CH_2Cl_2 and Et_3N were distilled from CaH_2 immediately prior to use. The reagent 3,5-dinitrobenzoyl chloride was purchased and used as received. The measurement of enantiomeric excesses (ee) was performed on an Agilent 1200 HPLC Series system with a Daicel Chiralcel OJ-H column. Optical rotations were determined by a Rudolph AUTOPOL-IV polarimeter at 25 ^{o}C. ^{1}H and ^{13}C NMR spectra were recorded at 500 or 125 MHz on a Bruker Ascend™ 500MHz spectrometer, respectively. Tetramethylsilane (0.00 ppm) was used as internal standard for ^{1}H NMR and $CDCl_3$ (77.16 ppm) for ^{13}C NMR. High resolution mass (HRMS) data were taken on a Waters LCT Premier™ with an ESI mass spectrometer.

2. Synthesis of 3,5-dinitrobenzoates (*R*)- and (*S*)-11

Scheme S1. Synthesis of 3,5-dinitrobenzoates (*R*)- and (*S*)-**11**.

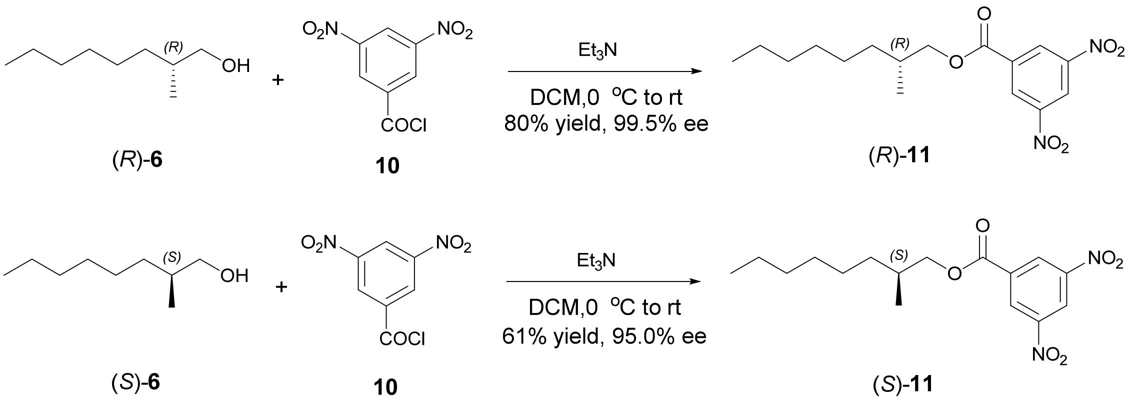

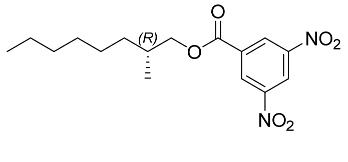

(*R*)-2-methyloctanyl 3,5-dinitrobenzoate ((*R*)-11) (new compound)[1]

(*R*)-2-Methyloctan-1-ol ((*R*)-**6**) (0.0020 g, 0.14 mmol) was dissolved in anhydrous CH_2Cl_2 (2 mL) at room temperature. The solution was cooled to 0 °C, then Et_3N (0.031g, 0.306 mmol) and 3,5-dinitrobenzoyl chloride (**10**) (0.068 g, 0.295 mmol) in dry CH_2Cl_2 (2 mL) were added. After the reaction

mixture was allowed to warm to room temperature and stirred for 12 h, monitored by thin-layer chromatography (petroleum ether/ethyl acetate = 5:1), it was quenched with saturated aqueous NH_4Cl (4 mL). The organic phase was separated and the aqueous phase was extracted with CH_2Cl_2 (3 × 4 mL). The combined CH_2Cl_2 extracts and the organic phase were d washed with brine (18 mL), dried over anhydrous Na_2SO_4, and concentrated. The final column chromatography on silica gel (petroleum ether/ethyl acetate 50:1) afforded (*R*)-2-methyloctanyl 3,5-dinitrobenzoate ((*R*)-10) (0.038 g, 80% yield, 99.5% ee) as a yellow solid. The ee was determined by chiral HPLC (Daicel Chiralcel OJ-H column, 254 nm, *n*-hexane/isopropanol = 99.5:0.5, 1.0 mL/min, major t_r = 26.994 min (*R*), minor t_r = 30.787 min (*S*)). R_f = 0.75 (petroleum ether/ethyl acetate = 5:1). $[\alpha]_D^{25}$ = +9.05 (c 1.33, $CHCl_3$). ^1H NMR (500 MHz, $CDCl_3$ δ 9.28 – 9.27 (m, 1H), 9.21 – 9.20 (m, 2H), 4.39 (dd, *J* = 10.7, 5.8 Hz, 1H), 4.29 (dd, *J* = 10.7, 7.0 Hz, 1H), 2.10 – 2.01 (m, 1H), 1.55 – 1.45 (m, 2H), 1.40 – 1.30 (m, 10H), 1.10 (d, *J* = 6.8 Hz, 3H), 0.93 (t, *J* = 6.6 Hz, 3H).^{13}C NMR (126 MHz, $CDCl_3$) δ 162.69, 148.80, 134.30, 129.50, 122.43, 71.95, 33.47, 32.74, 31.91, 29.56, 26.89, 22.75, 17.10, 14.20. HRMS (ESI) calcd for $C_{16}H_{21}N_2O_6$ $[M-H]^+$ 337.13941, found 337.13893.

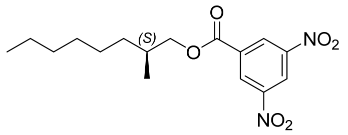

(*S*)-2-methyloctanyl 3,5-dinitrobenzoate ((*S*)-11) (new compound)

According to the similar procedure for 3,5-dinitrobenzoate (*R*)-**11**, the esterification of (*S*)-2-methyloctan-1-ol (*S*)-**6** (0.0020 g, 0.14 mmol) with 3,5-dinitrobenzoyl chloride (**10**) (0.034 g, 0.147 mmol) afforded (*S*)-2-methyloctanyl 3,5-dinitrobenzoate ((*S*)-**11**) (0.029 g, 61% yield, 95.0% ee) as a yellow solid. The ee was determined by chiral HPLC (Daicel Chiralcel OJ-H column, 254 nm, *n*-hexane/isopropanol = 99.5:0.5, 1.0 mL/min, minor t_r = 29.242 min (*R*), major t_r = 31.092 min (*S*)). R_f = 0.75 (petroleum ether/ethyl acetate = 5:1). $[\alpha]_D^{25}$ = -5.65 (c 2.55, $CHCl_3$). ^1H NMR (500 MHz, $CDCl_3$) δ 9.19 – 9.18 (m, 1H), 9.11 – 9.10 (m, 2H), 4.30 (dd, *J* = 10.7, 5.8 Hz, 1H), 4.20 (dd, *J* = 10.7, 7.0 Hz, 1H), 2.00 – 1.91 (m, 1H), 1.45 – 1.35 (m, 1H), 1.32 – 1.20 (m, 8H), 1.00 (d, *J* = 6.8 Hz, 3H), 0.83 (d, *J* = 7.1 Hz, 3H). ^{13}C NMR (126 MHz, $CDCl_3$) δ 162.70, 148.81, 134.31, 129.50, 122.43, 71.95, 33.48, 32.75, 31.91, 29.57, 26.90, 22.76, 17.10, 14.21. HRMS (ESI) calcd for $C_{16}H_{23}N_2O_6$ $[M+H]^+$ 339.15506, found 339.15421.

3. ^{1}H, ^{13}C NMR Spectra of the Products

Figure S1. ^{1}H NMR Spectrum of (4*R*,5*S*)-4-methyl-3-octanoyl-5-phenyloxazolidin-2-one ((4*R*,5*S*)-**4**) (500 MHz, $CDCl_3$)

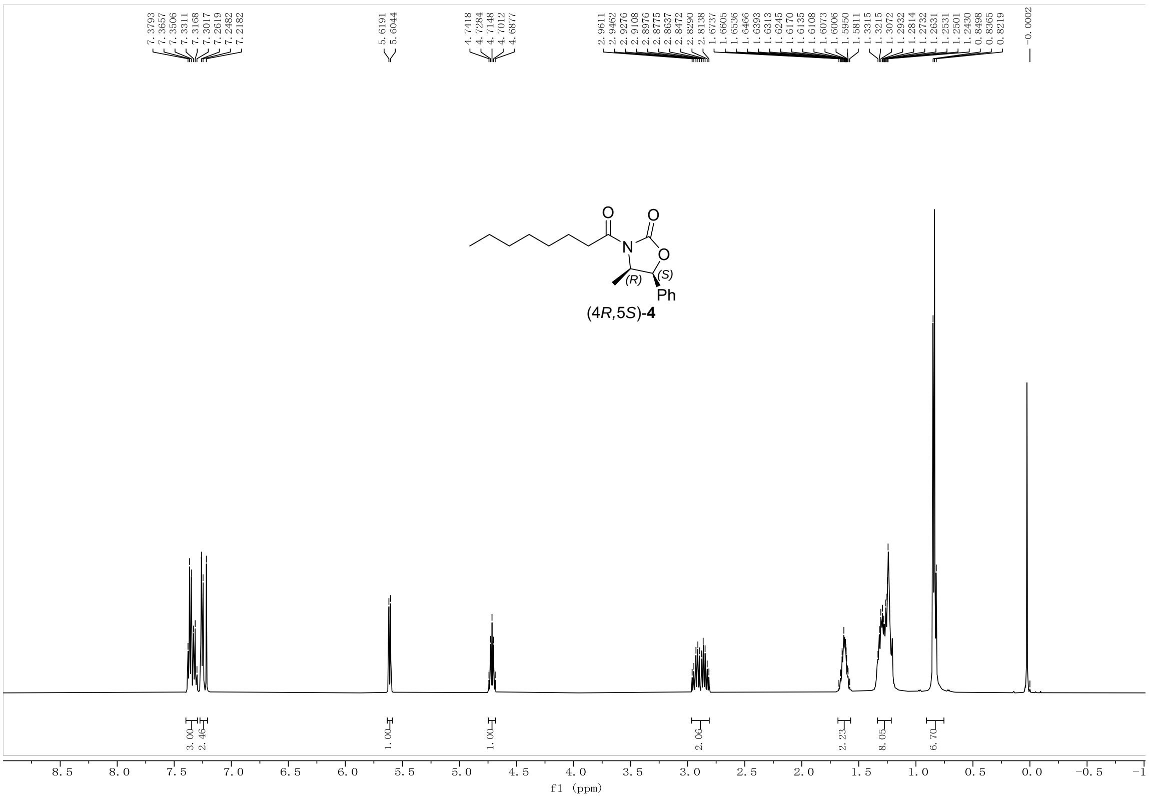

Figure S2. ^{13}C NMR Spectrum of (4*R*,5*S*)-4-methyl-3-octanoyl-5-phenyloxazolidin-2-one ((4*R*,5*S*)-**4**) (126 MHz, $CDCl_3$)

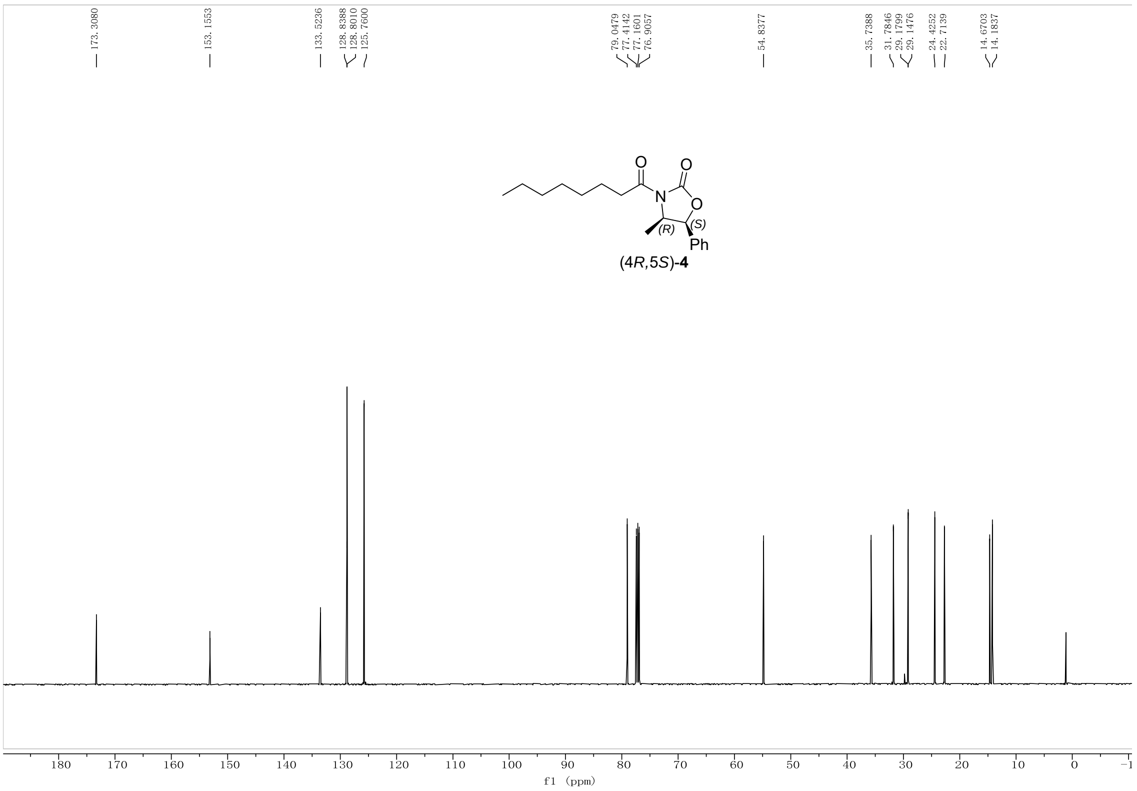

Figure S3. ^{1}H NMR Spectrum of (4*R*,5*S*)-4-methyl-3-((*R*)-2-methyloctanoyl)-5-phenyloxazolidin-2-one ((4*R*,5*S*,2′*R*)-**5**) (500 MHz, $CDCl_3$)

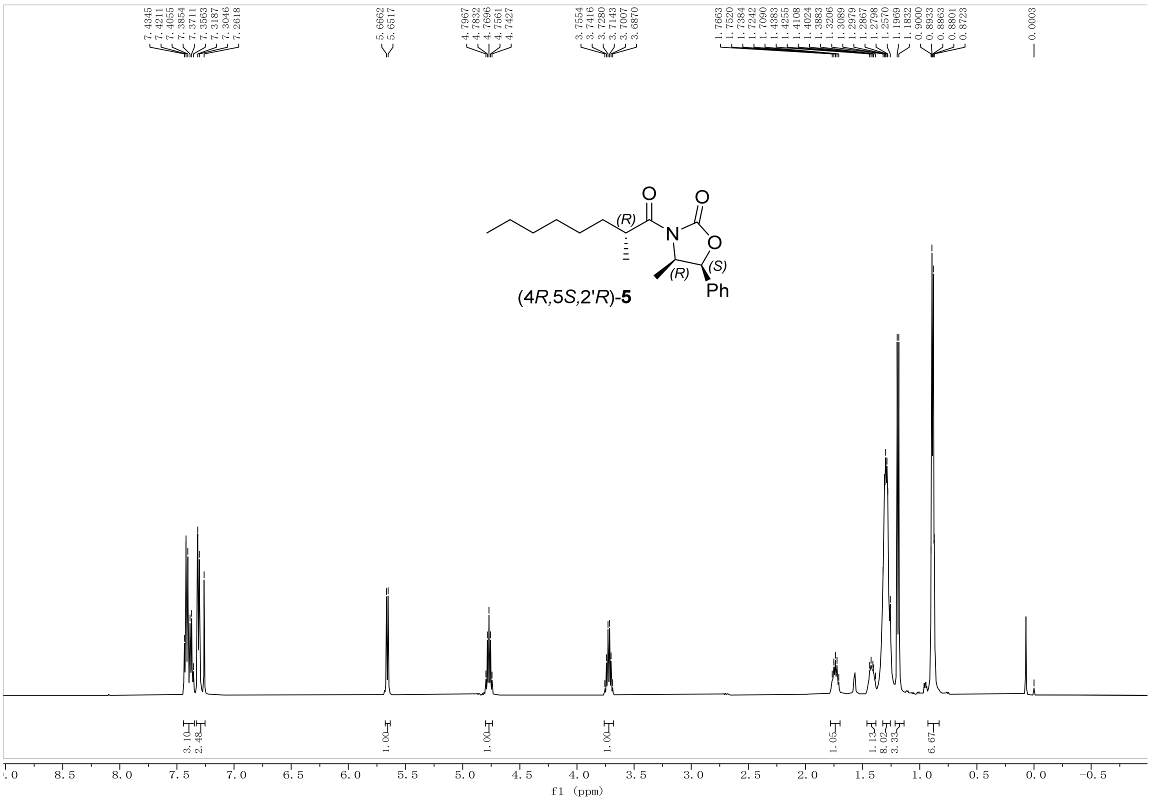

Figure S4. ^{13}C NMR Spectrum of (4*R*,5*S*)-4-methyl-3-((*R*)-2-methyloctanoyl)-5-phenyloxazolidin-2-one ((4*R*,5*S*,2′*R*)-**5**) (126 MHz, $CDCl_3$)

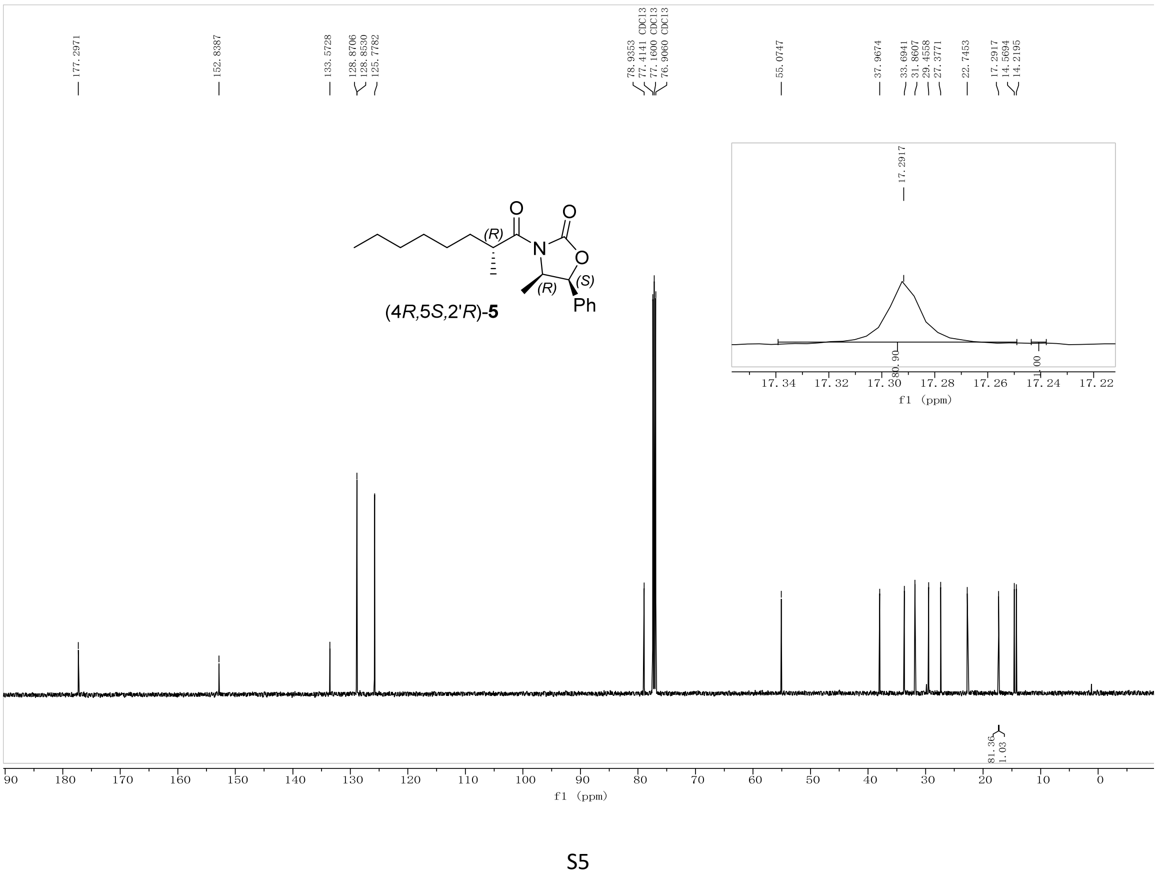

Figure S5. ^{1}H NMR Spectrum of (*R*)-2-methyloctan-1-ol ((*R*)-**6**) (500 MHz, $CDCl_3$)

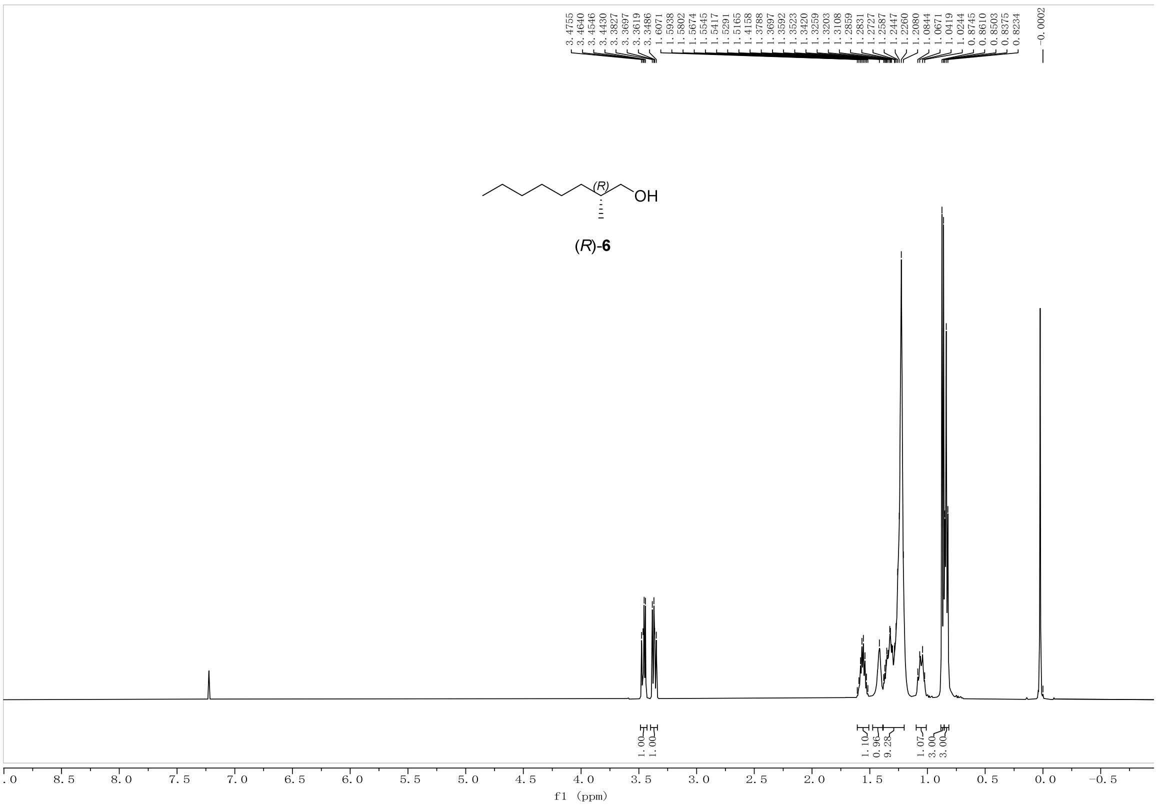

Figure S6. ^{13}C NMR Spectrum of (*R*)-2-methyloctan-1-ol ((*R*)-**6**) (126 MHz, $CDCl_3$)

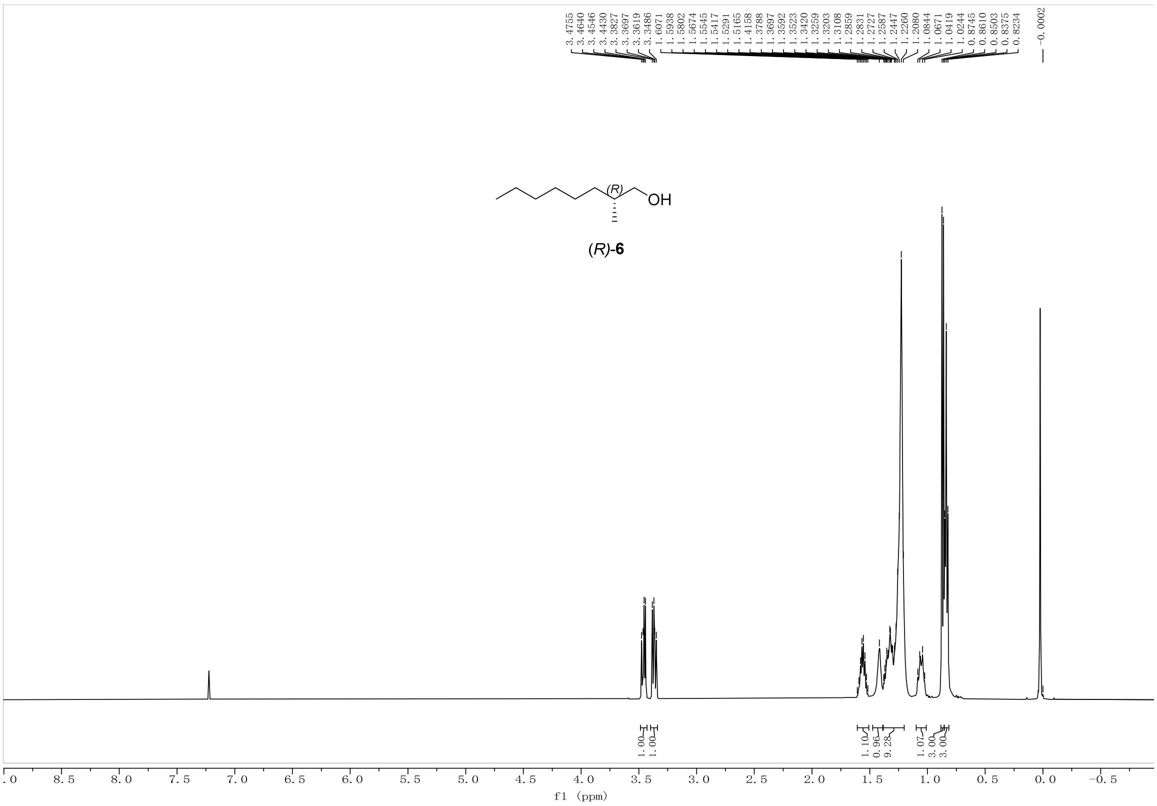

Figure S7. ^{1}H NMR Spectrum of (4*S*,5*R*)-4-methyl-3-octanoyl-5-phenyloxazolidin-2-one ((4*S*,5*R*)-**4**) (500 MHz, $CDCl_3$)

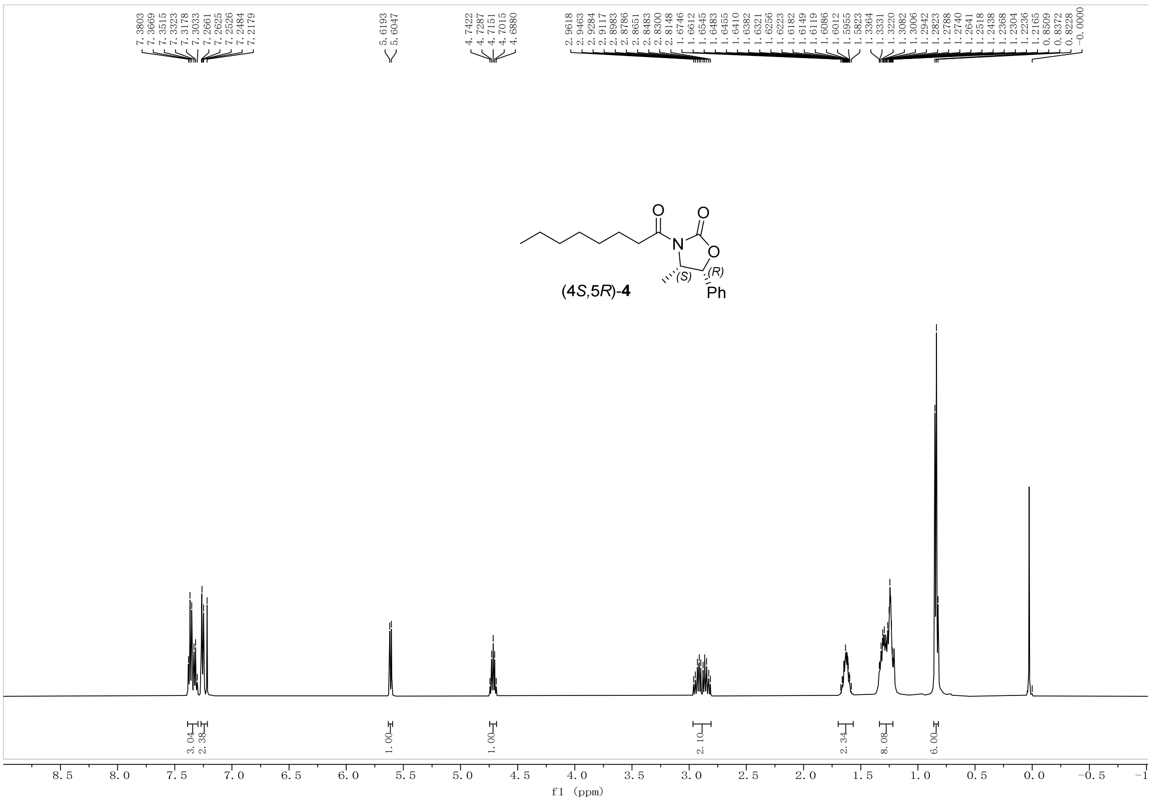

Figure S8. ^{13}C NMR Spectrum of (4*S*,5*R*)-4-methyl-3-octanoyl-5-phenyloxazolidin-2-one ((4*S*,5*R*)-**4**) (126 MHz, $CDCl_3$)

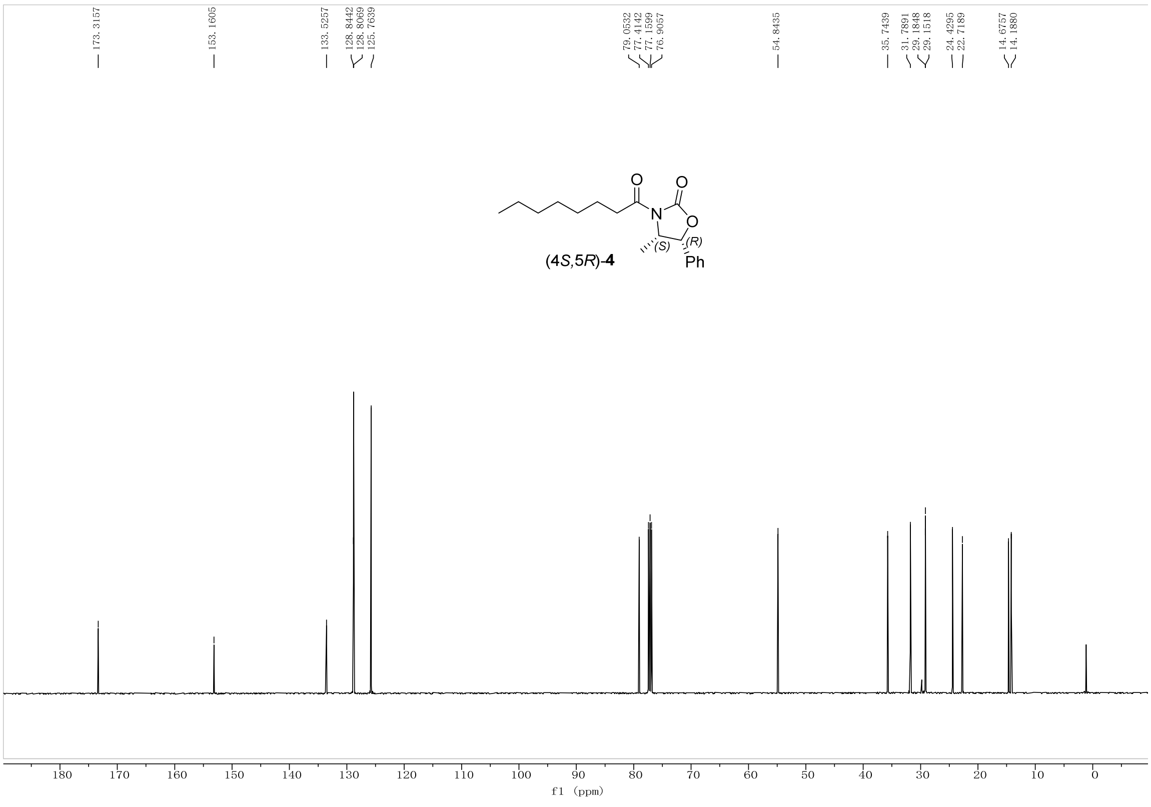

Figure S9. ^{1}H NMR Spectrum of (4*S*,5*R*)-4-methyl-3-((*S*)-2-methyloctanoyl)-5-phenyloxazolidin-2-one((4*S*,5*R*,2′*S*)-**5**) (500 MHz, $CDCl_3$)

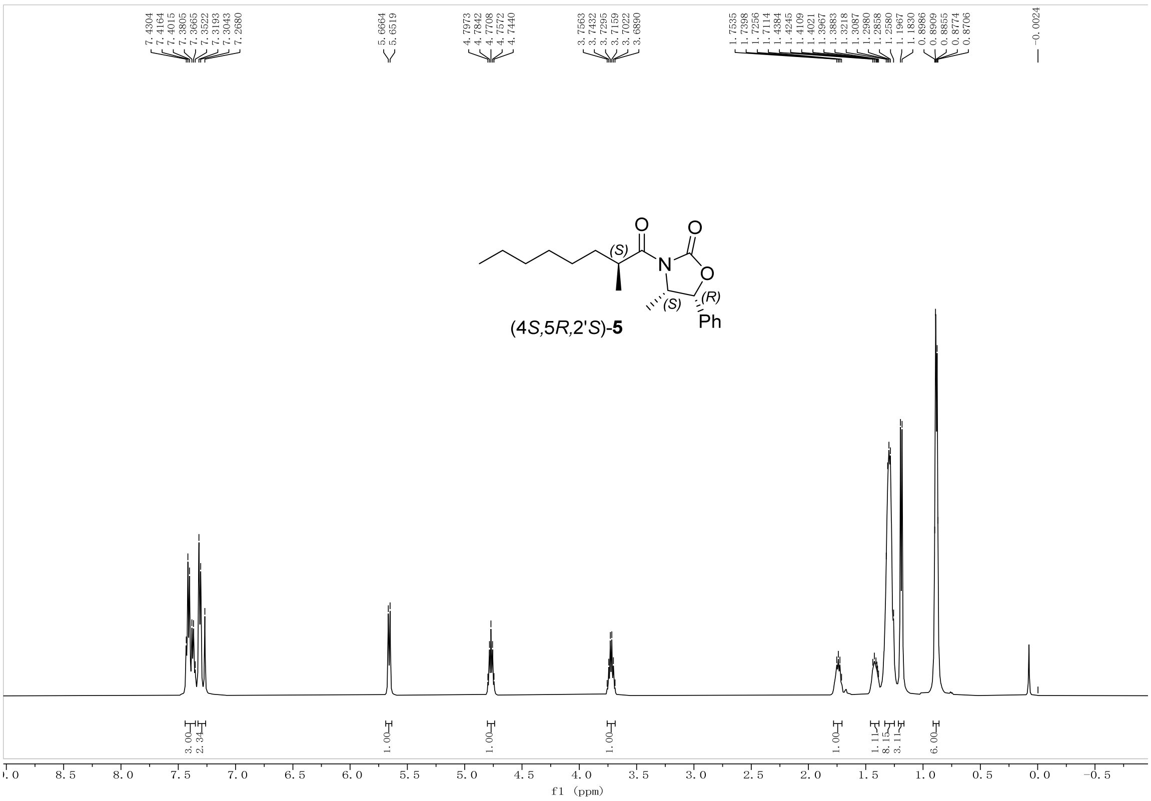

Figure S10. ^{13}C NMR Spectrum of (4*S*,5*R*)-4-methyl-3-((*S*)-2-methyloctanoyl)-5-phenyloxazolidin-2-one((4*S*,5*R*,2′*S*)-**5**) (126 MHz, $CDCl_3$)

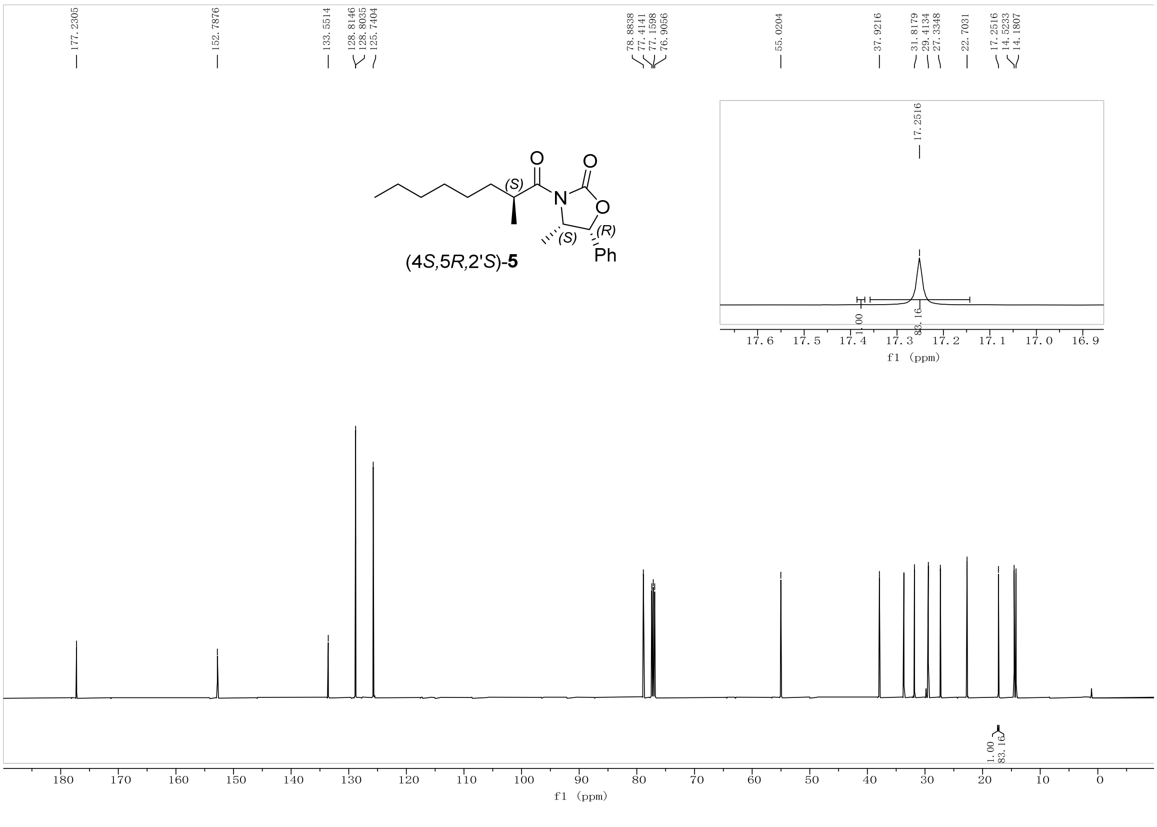

Figure S11. ^{1}H NMR Spectrum of (*S*)-2-methyloctan-1-ol ((*S*)-**6**) (500 MHz, $CDCl_3$)

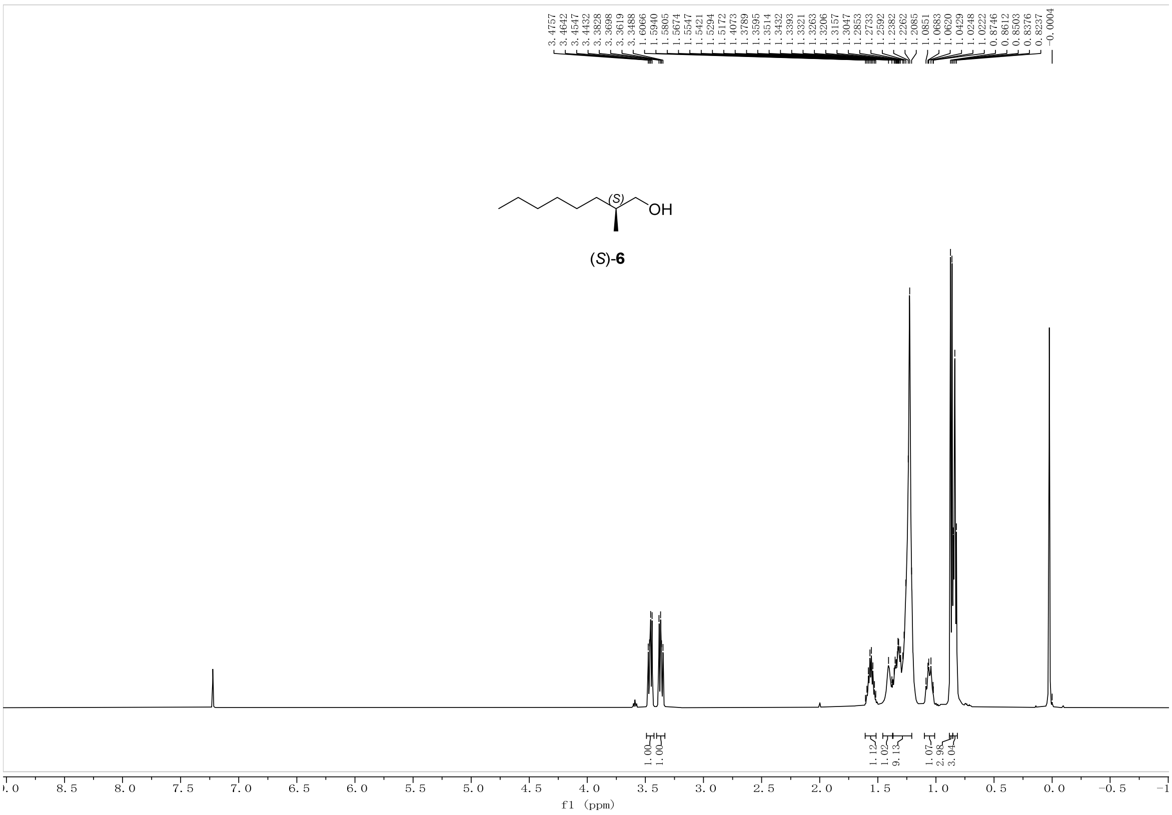

Figure S12. ^{13}C NMR Spectrum of (*S*)-2-methyloctan-1-ol ((*S*)-**6**) (126 MHz, $CDCl_3$)

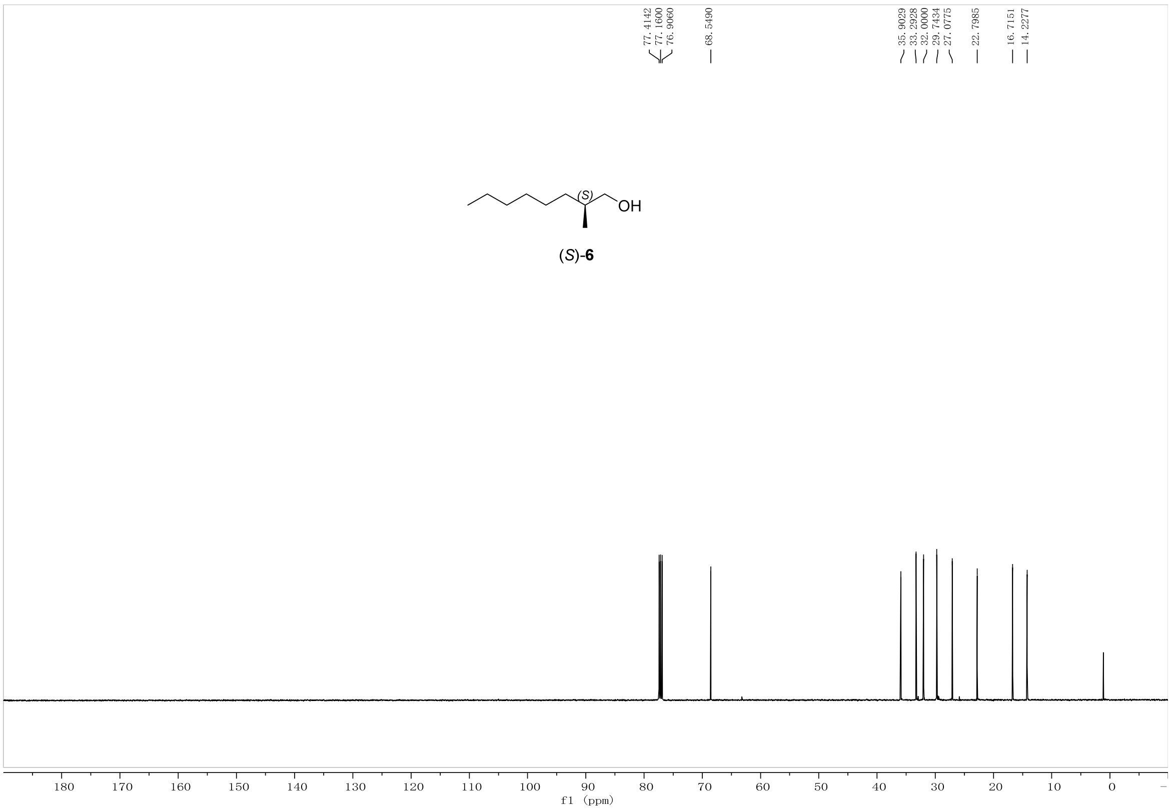

Figure S13. ^{1}H NMR Spectrum of (*R*)-1-bromo-2-methyloctane ((*R*)-**7**) (500 MHz, CDCl$_3$)

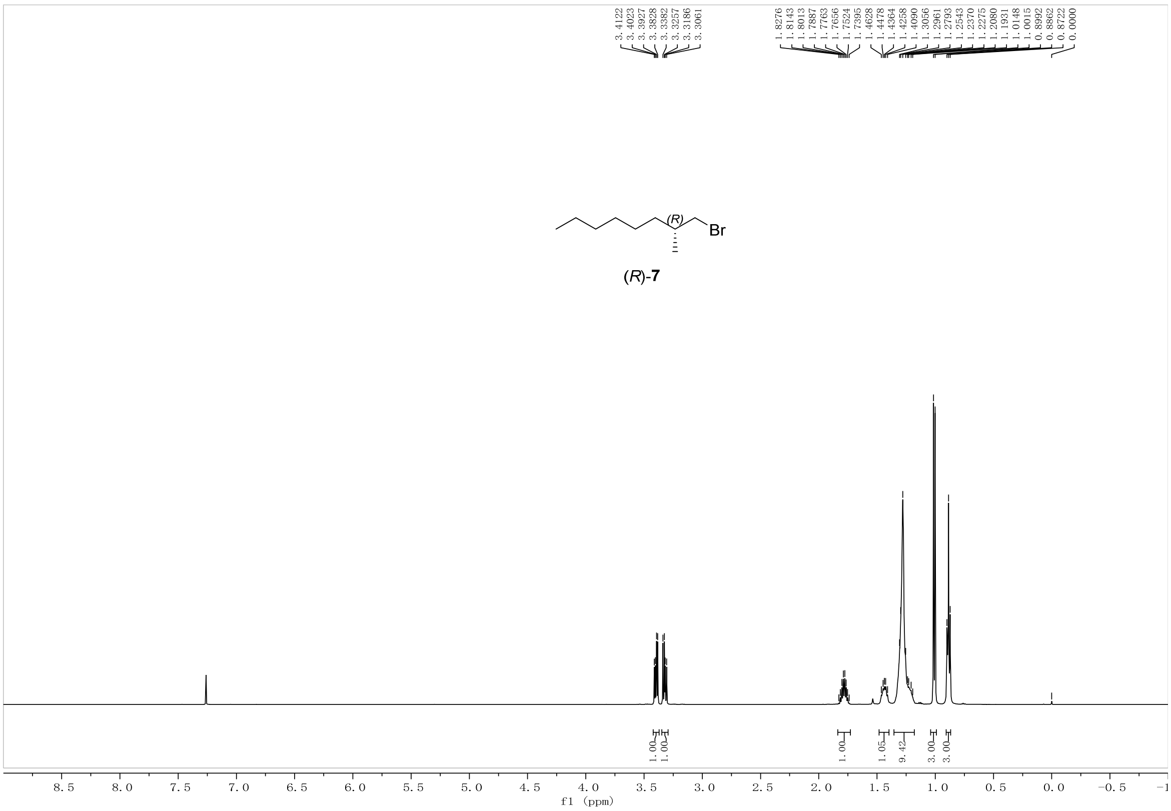

Figure S14. ^{13}C NMR Spectrum of (*R*)-1-bromo-2-methyloctane ((*R*)-**7**) (126 MHz, CDCl$_3$)

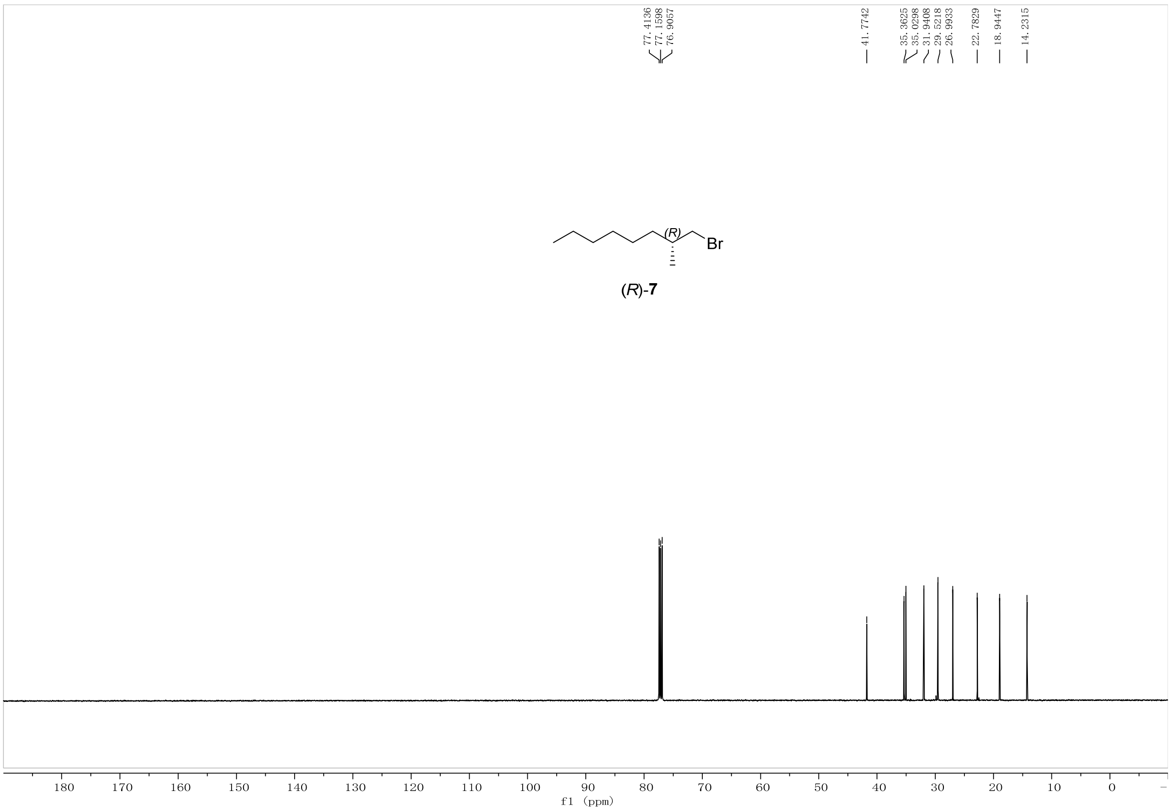

Figure S15. ^{1}H NMR Spectrum of (*R*)-7-methyltricos-8-ene ((*R*)-**9**) (500 MHz, $CDCl_3$)

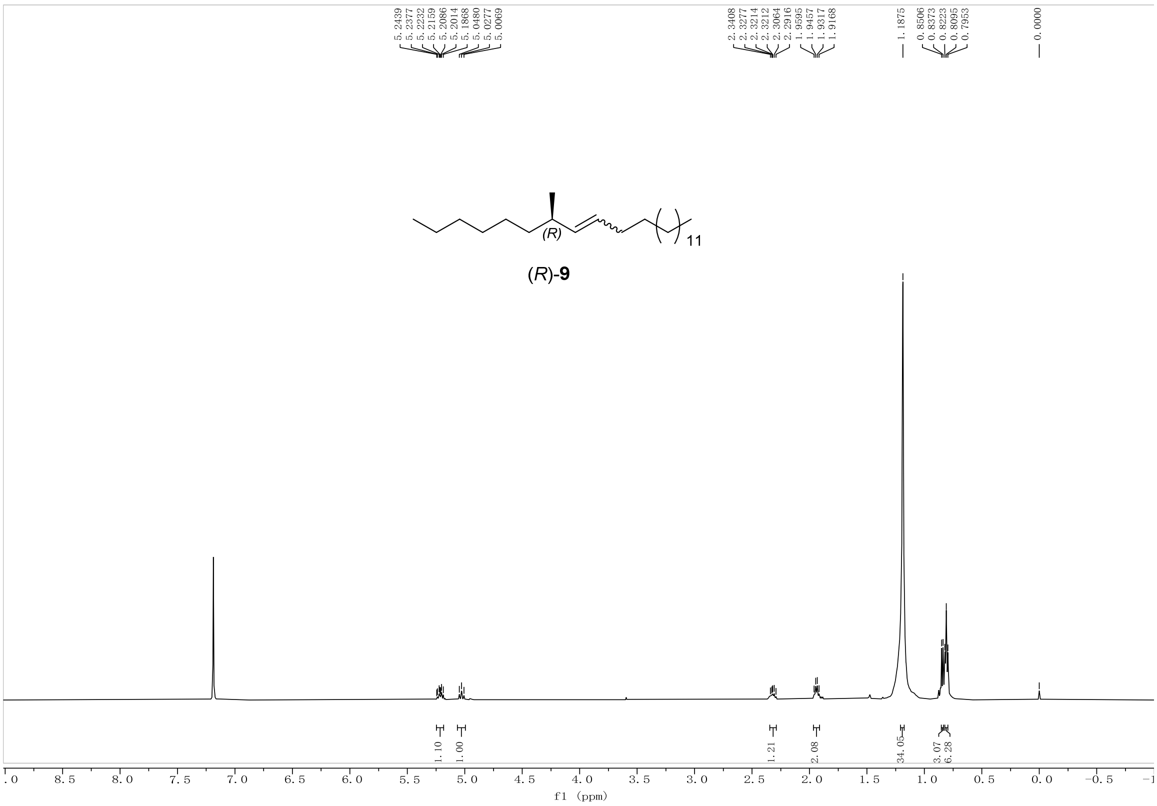

Figure S16. ^{13}C NMR Spectrum of (*R*)-7-methyltricos-8-ene ((*R*)-**9**) (126 MHz, $CDCl_3$)

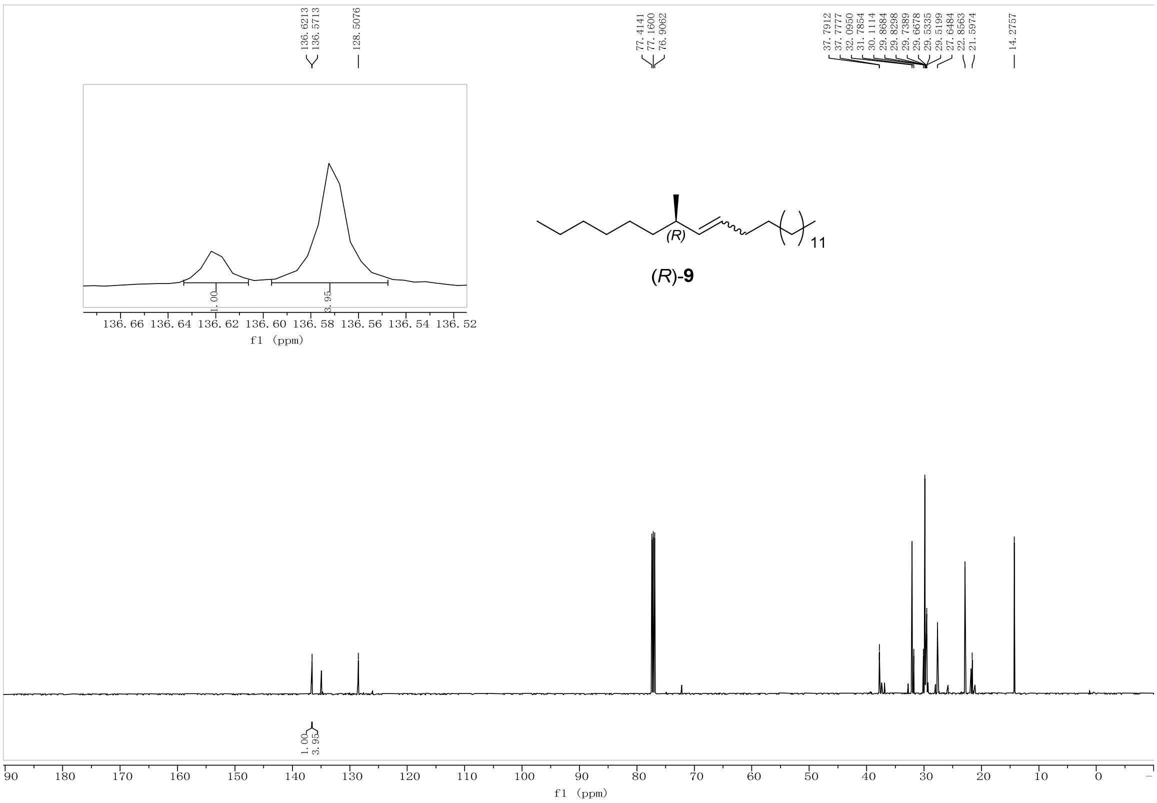

Figure S17. ^{1}H NMR Spectrum of (*R*)-7-methyltricosane ((*R*)-**1**) (500 MHz, $CDCl_3$)

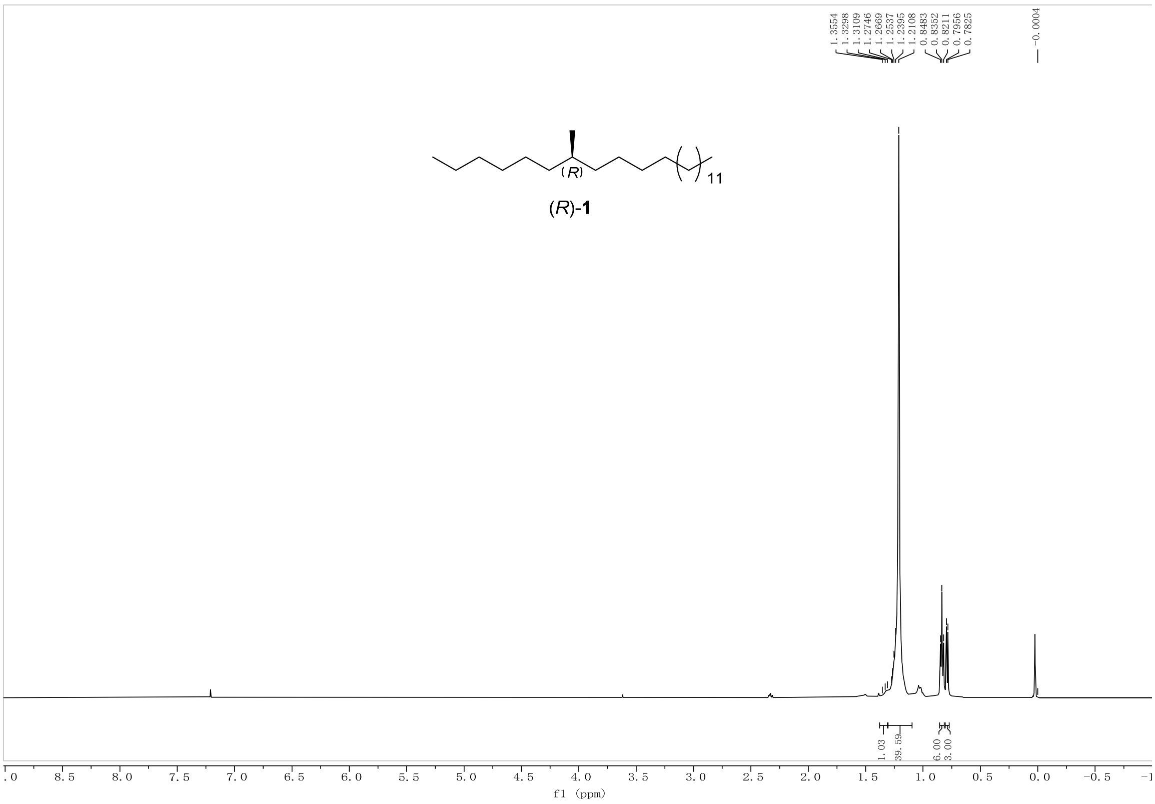

Figure S18. ^{13}C NMR Spectrum of (*R*)-7-methyltricosane ((*R*)-**1**) (126 MHz, $CDCl_3$)

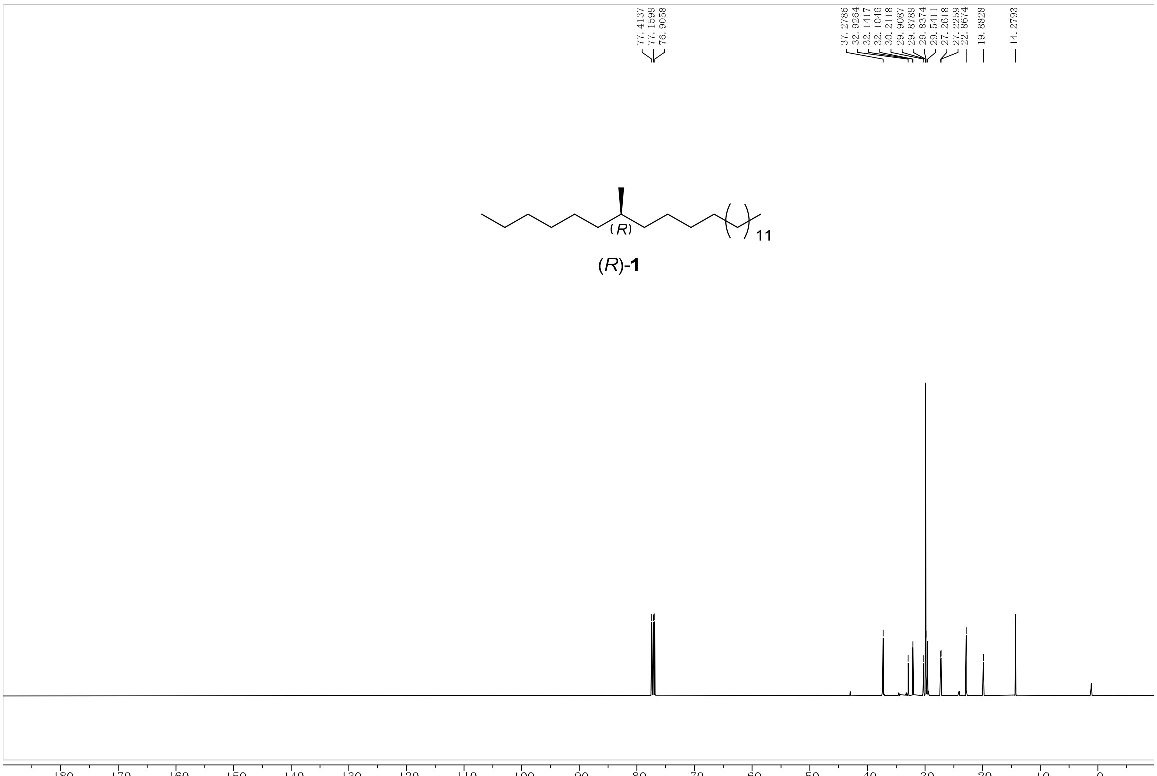

Figure S19. ^{1}H NMR Spectrum of (*S*)-1-bromo-2-methyloctane ((*S*)-**7**) (500 MHz, $CDCl_3$)

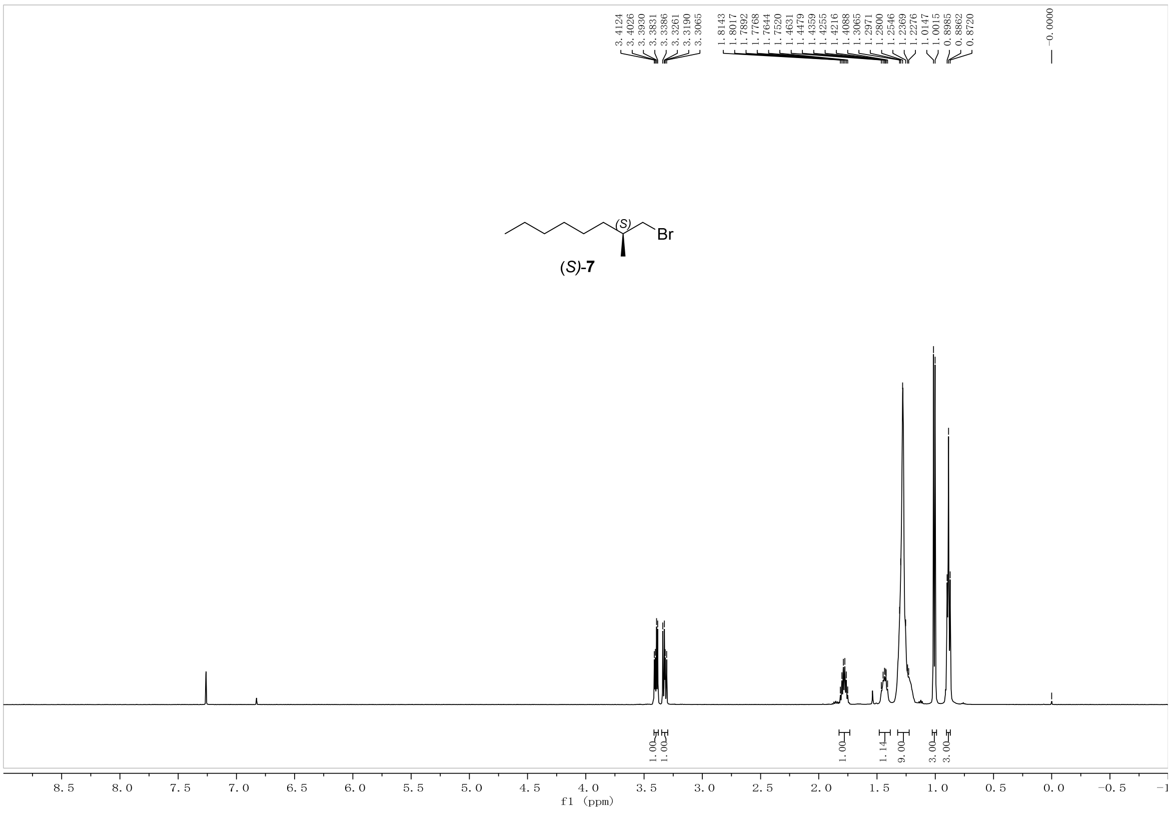

Figure S20. ^{13}C NMR Spectrum of (*S*)-1-bromo-2-methyloctane((*S*)-**7**) (126 MHz, $CDCl_3$)

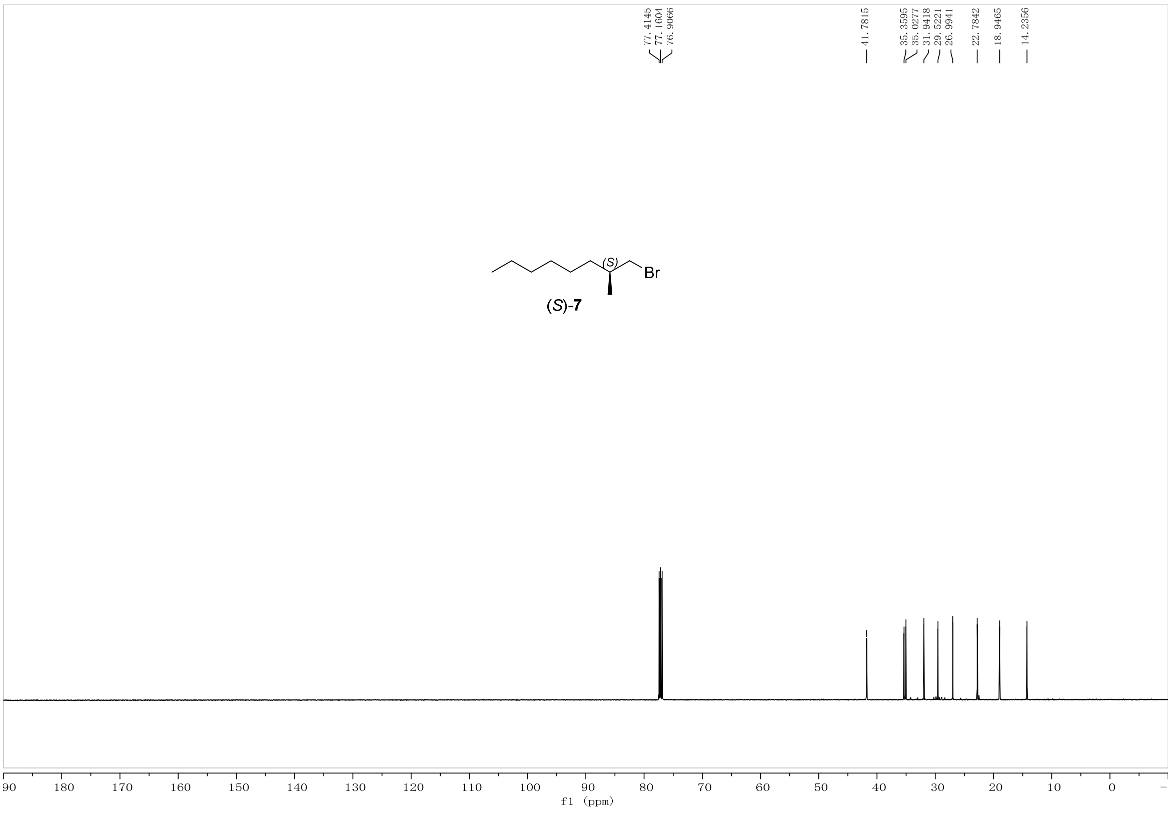

Figure S21. ^{1}H NMR Spectrum of (*S*)-7-methyltricos-8-ene ((*S*)-**9**) (500 MHz, $CDCl_3$)

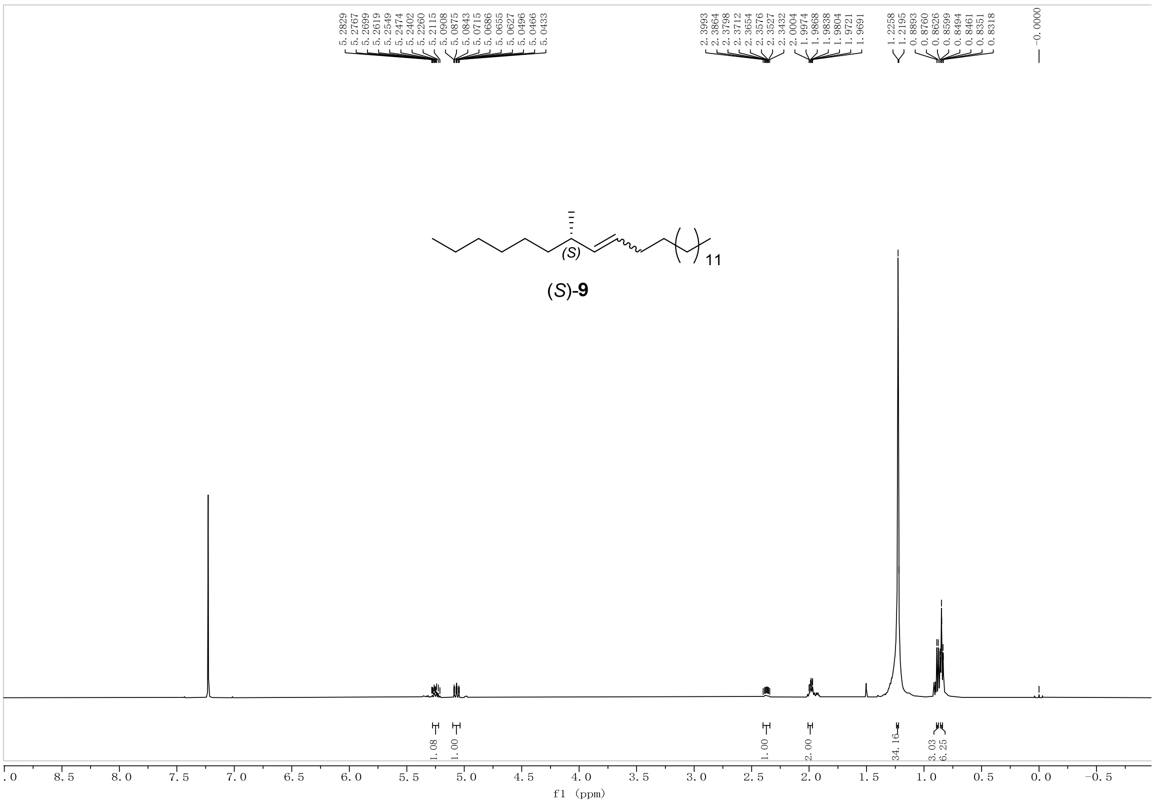

Figure S22. ^{13}C NMR Spectrum of (*S*)-7-methyltricos-8-ene ((*S*)-**9**) (126 MHz, $CDCl_3$)

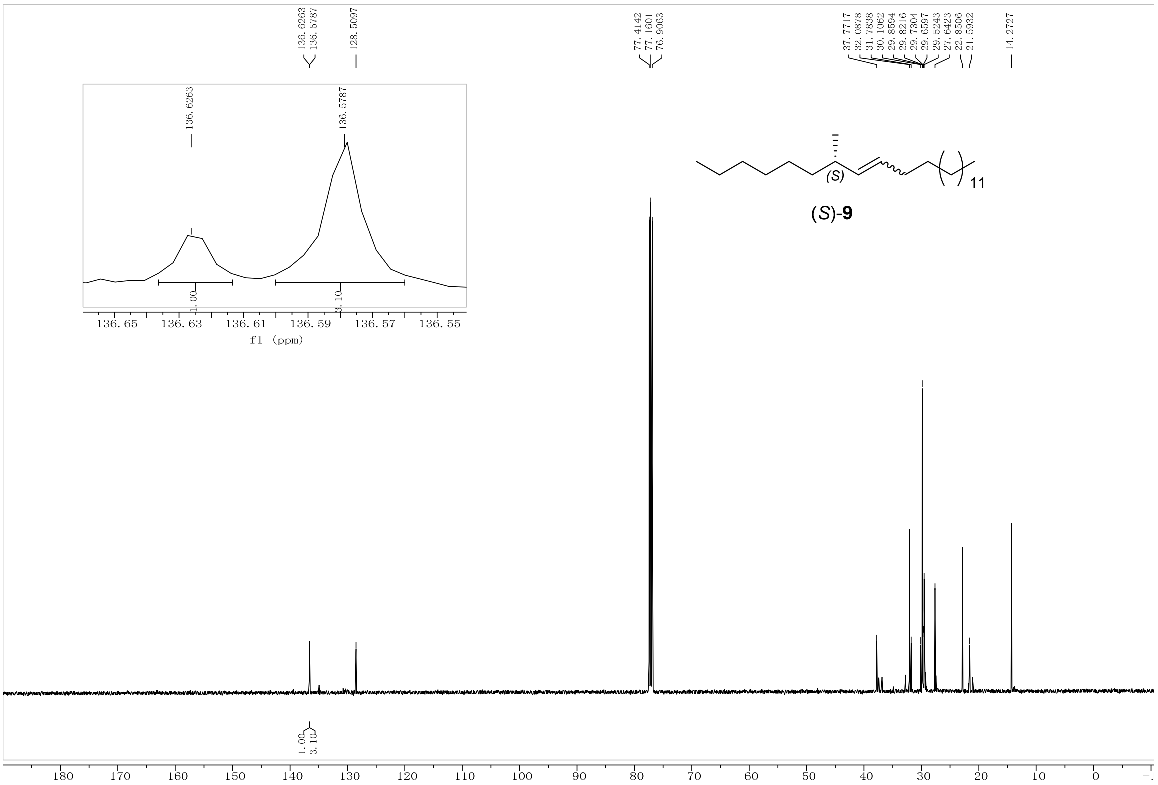

Figure S23. ^{1}H NMR Spectrum of (*S*)-7-methyltricosane ((*S*)-**1**) (500 MHz, CDCl$_3$)

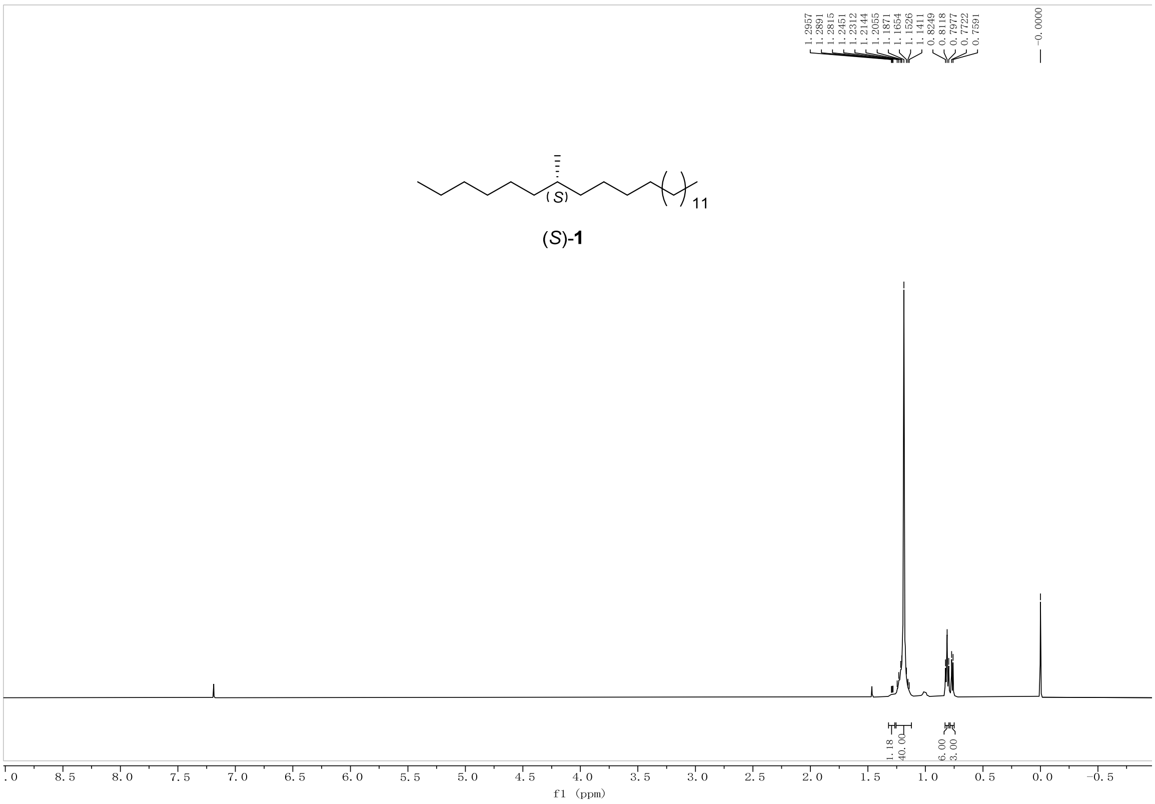

Figure S24. ^{13}C NMR Spectrum of (*S*)-7-methyltricosane ((*S*)-**1**) (126 MHz, CDCl$_3$)

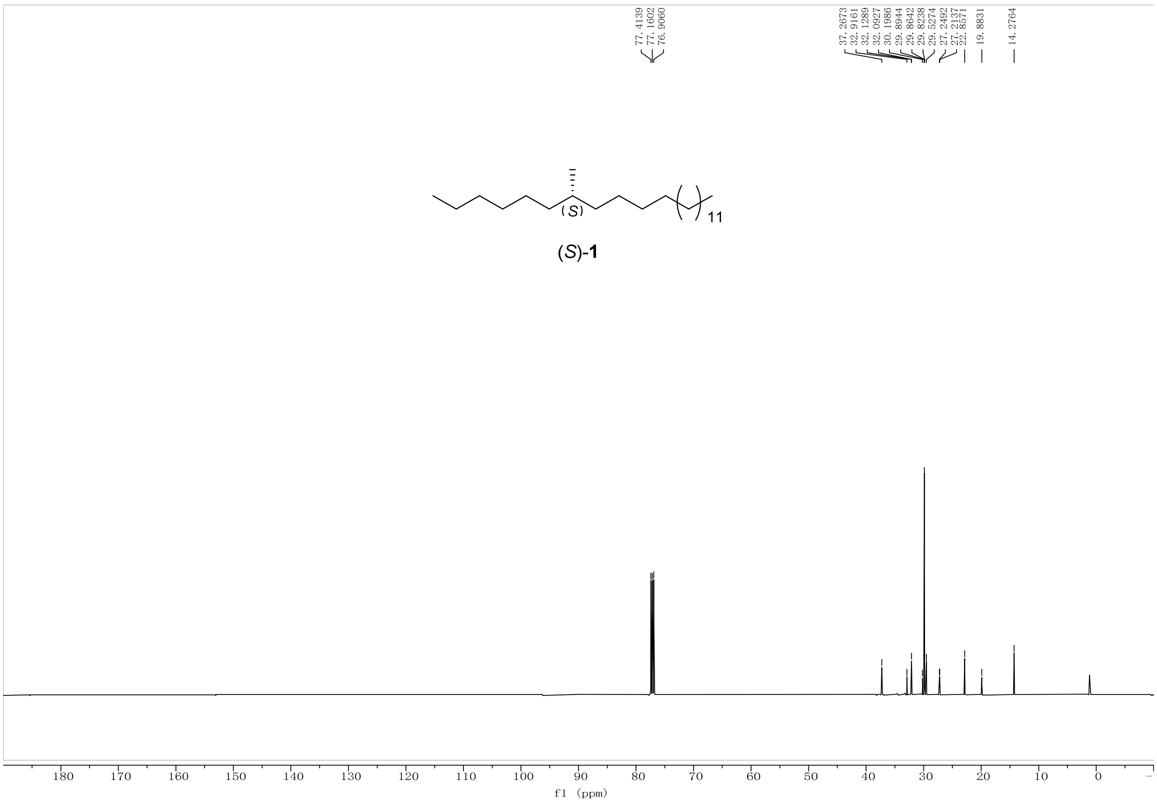

Figure S25. ^{1}H NMR Spectrum of (*R*)-2-methyloctanyl 3,5-dinitrobenzoate ((*R*)-**11**) (500 MHz, $CDCl_3$)

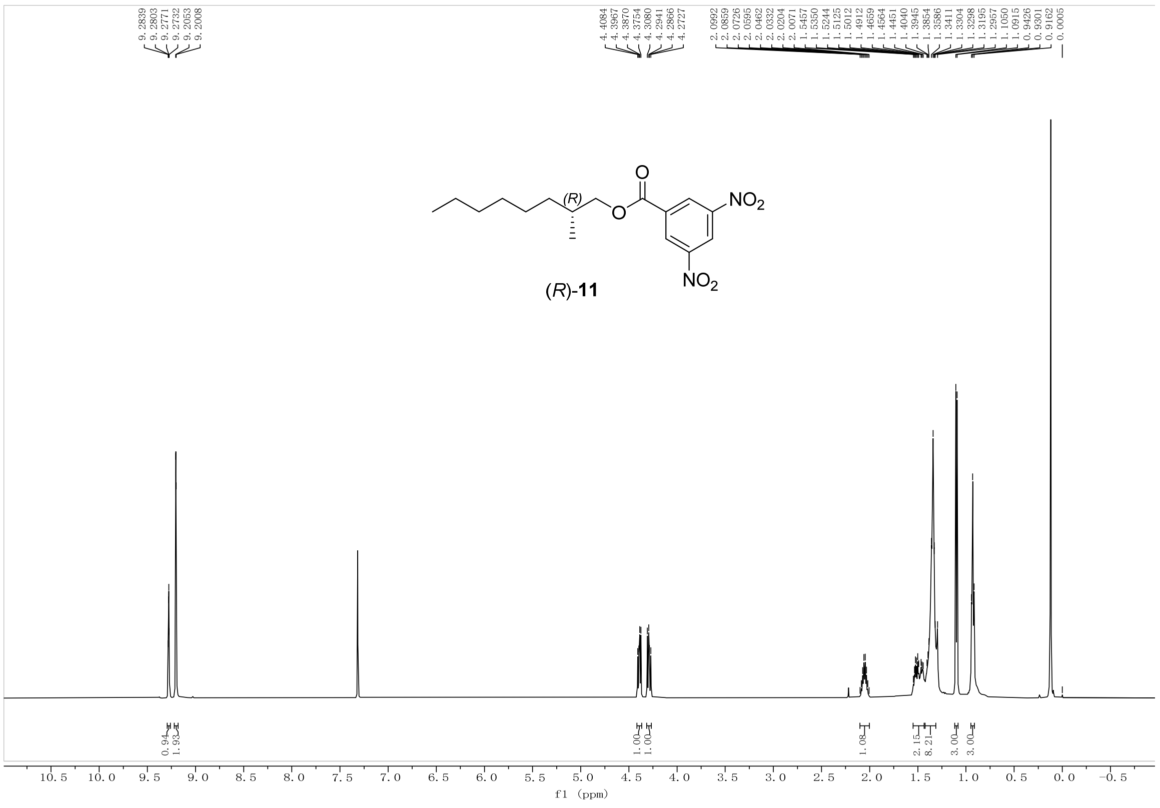

Figure S26. ^{13}C NMR Spectrum of (*R*)-2-methyloctanyl 3,5-dinitrobenzoate ((*R*)-**11**) (126 MHz, $CDCl_3$)

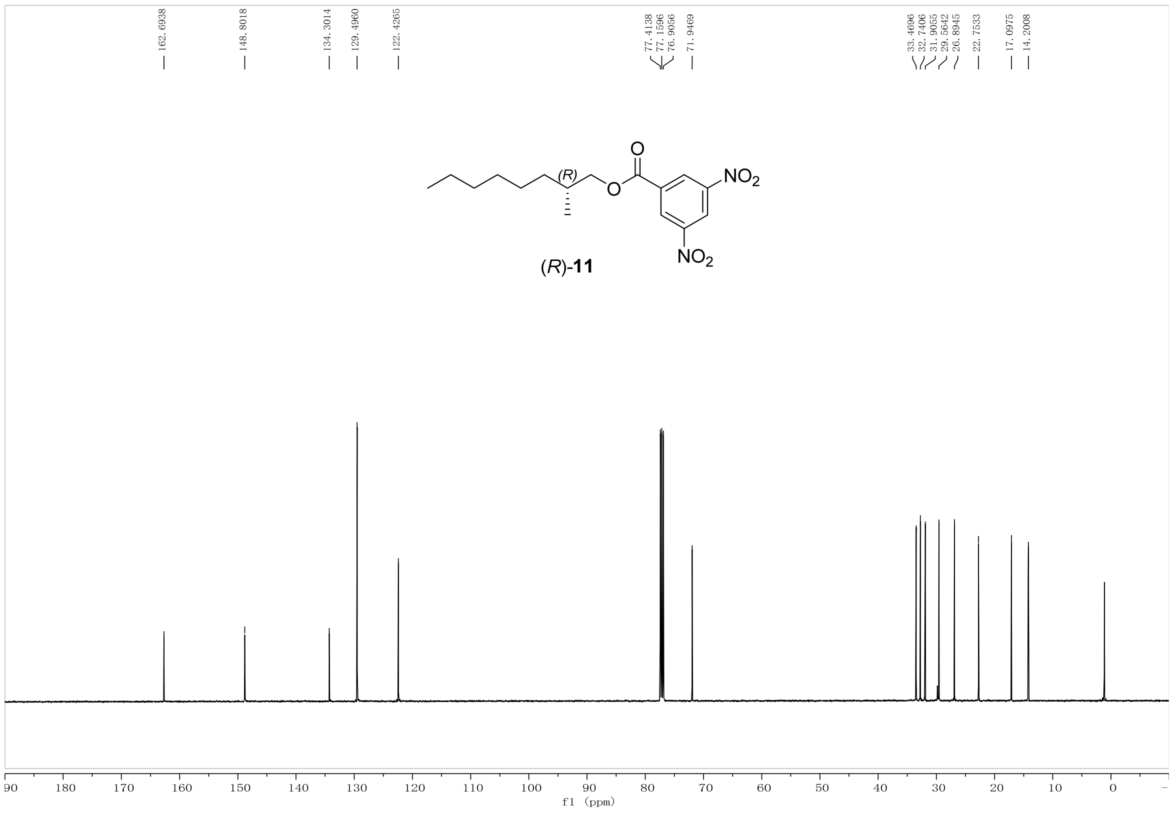

Figure S27. ^{1}H NMR Spectrum of (*S*)-2-methyloctanyl 3,5-dinitrobenzoate ((*S*)-**10**) (500 MHz, $CDCl_3$)

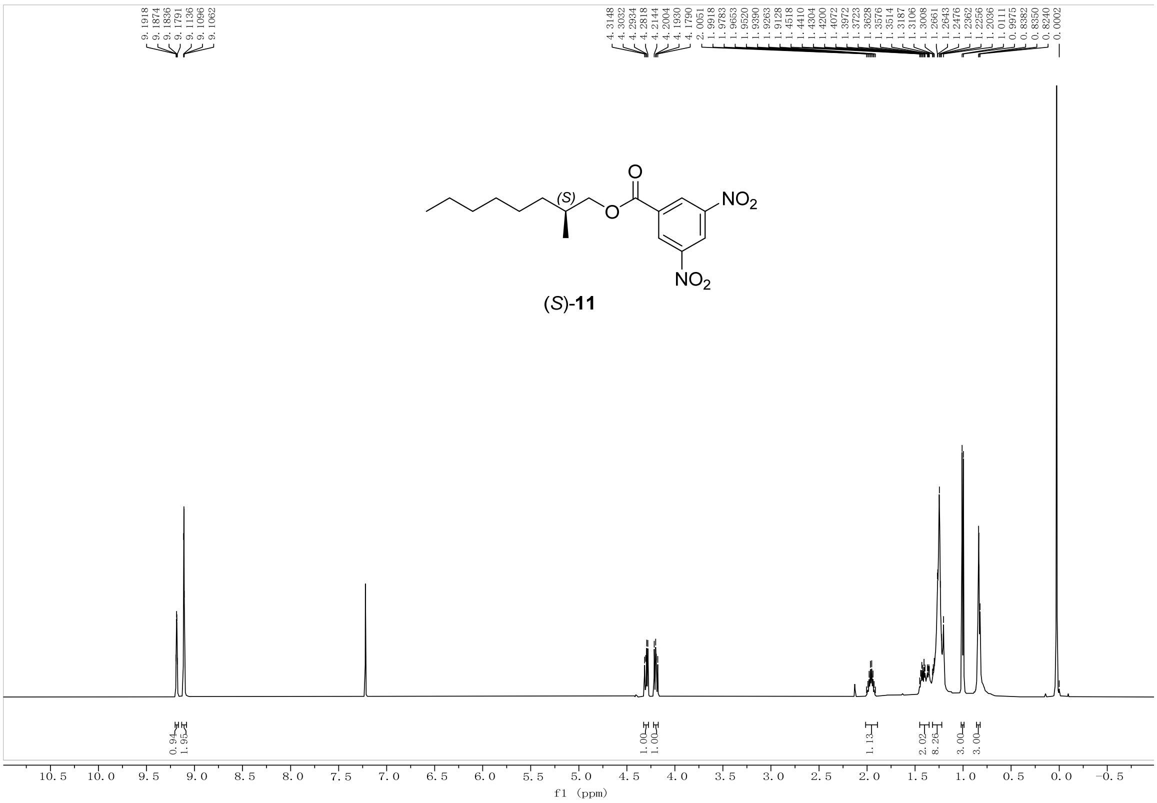

Figure S28. ^{13}C NMR Spectrum of (*S*)-2-methyloctanyl 3,5-dinitrobenzoate ((*S*)-**10**) (126 MHz, $CDCl_3$)

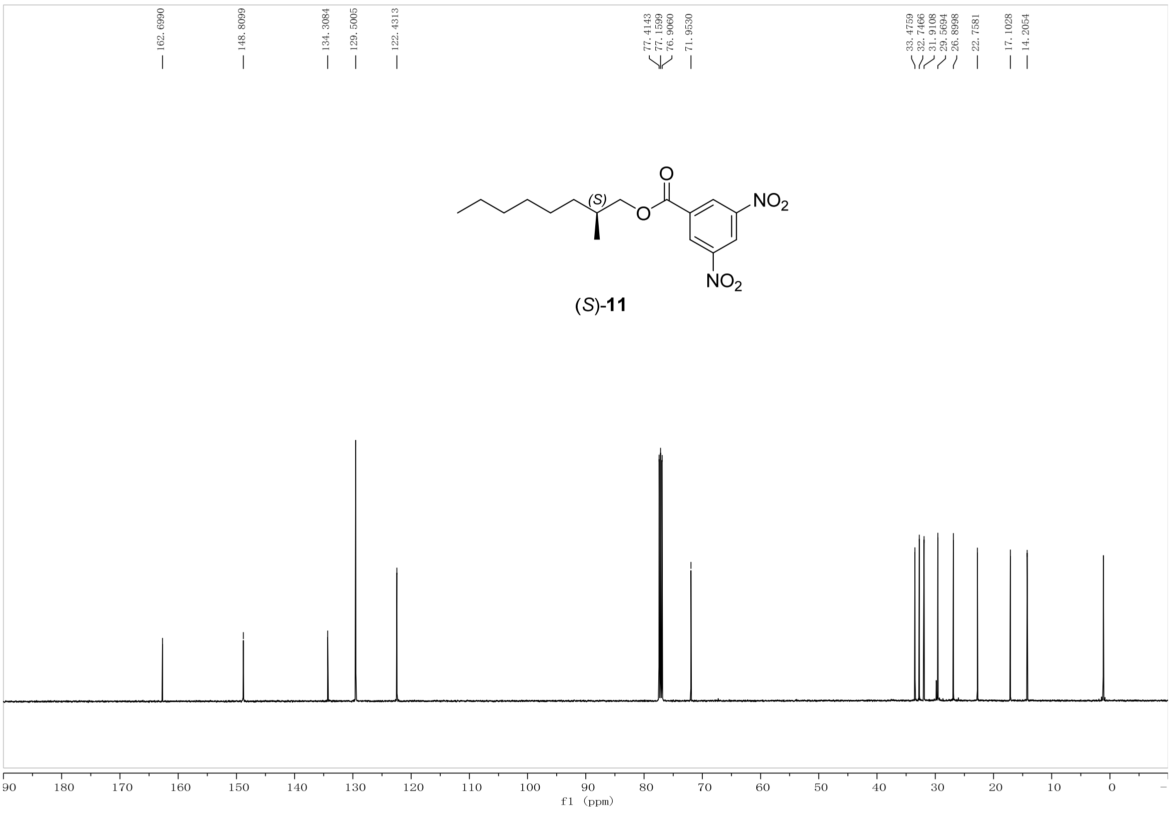

4. HPLC Chromatography of the Compounds

Figure S29. HPLC Chromatography of *rac*-2-methyloctanyl 3,5-dinitrobenzoate (*rac*-**11**) (Daicel Chiralcel OJ-H column; *n*-hexane/*i*-propanol = 99.5:0.5, 1.0 mL/min, 254 nm)

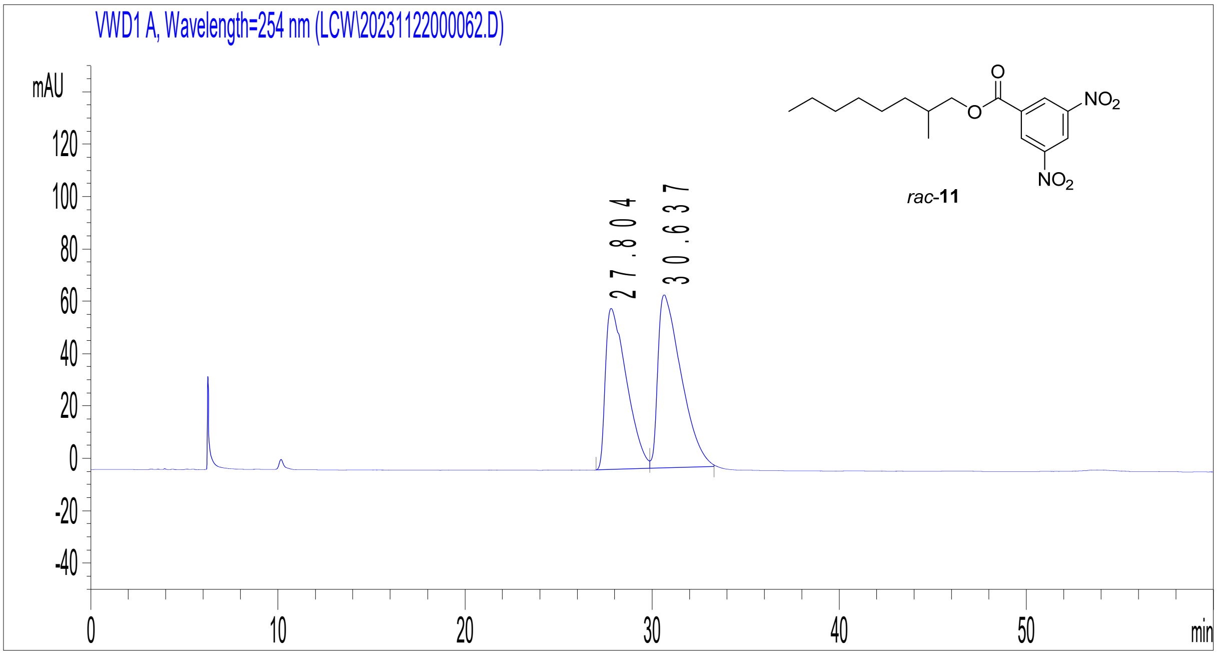

Peak #	RetTime [min]	Type	Width [min]	Area mAU*s	Height [mAU]	Area %
1	27.804	BB	1.1028	4848.18311	61.62437	45.6669
2	30.637	BB	1.2945	5768.22461	66.30396	54.3331

Figure S30. HPLC Chromatography of (*R*)-2-methyloctanyl 3,5-dinitrobenzoate ((*R*)-**11**) (Daicel Chiralcel OJ-H column; *n*-hexane/*i*-propanol = 99.5:0.5, 1.0 mL/min, 254 nm)

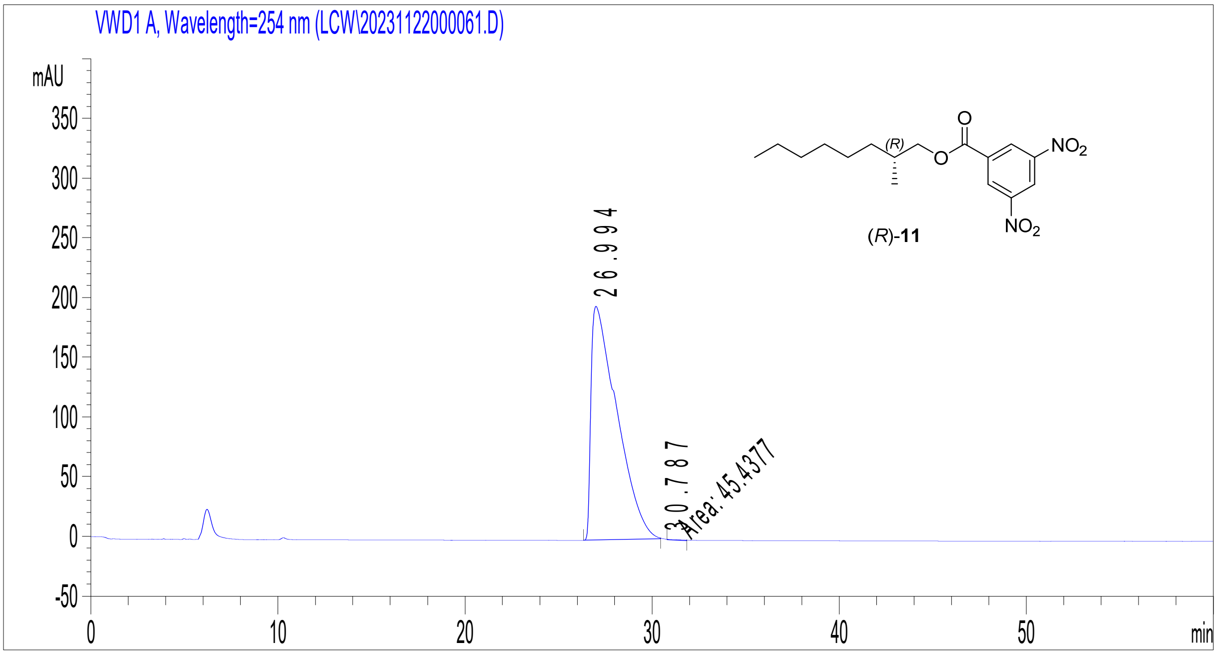

Peak #	RetTime [min]	Type	Width [min]	Area mAU*s	Height [mAU]	Area %
1	26.994	BB	1.3646	1.88781e4	195.89162	99.7599
2	30.787	MM	0.9616	45.43768	7.87551e-1	0.2401

Figure S31. HPLC Chromatography of *(S)-2*-methyloctanyl 3,5-dinitrobenzoate ((*S*)-**11**) (Daicel Chiralcel OJ-H column; *n*-hexane/*i*-propanol = 99.5:0.5, 1.0 mL/min, 254 nm)

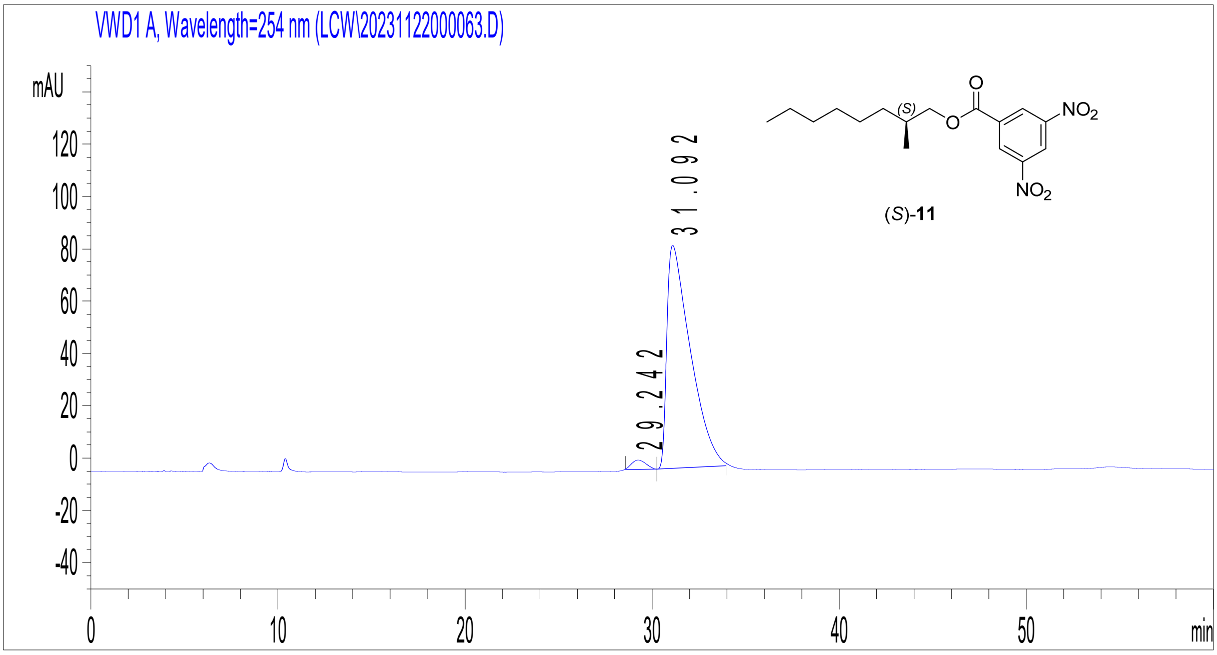

Peak #	RetTime [min]	Type	Width [min]	Area mAU*s	Height [mAU]	Area %
1	29.242	BB	0.8109	197.03148	3.64642	2.5762
2	31.092	BB	1.2470	7451.23682	85.36133	97.4238

5. References

(1) Leisering, S.; Riano, I.; Depken, C.; Gross, L. J.; Weber, M.; Lentz, D.; Zimmer, R.; Stark, C. B. W.; Breder, A.; Christmann, M. Synthesis of (+)-Greek tobacco lactone via a diastereoablative epoxidation and a selenium-catalyzed oxidative cyclization. *Org. Lett.* **2017**, *19*, 1478-1481.
